# Comparative differential cytotoxicity of clinically used SERMs in human cancer lines of different origin and its predictive molecular docking studies of key target genes involved in cancer progression and treatment responses

**DOI:** 10.1016/j.crphar.2021.100080

**Published:** 2021-12-31

**Authors:** Lakshmi S, Shanitha A, Shiny Dv, Rahul Bs, Saikant R, Shehna Sharaf, Abi Sa, Rajmohan G

**Affiliations:** a Corporate R&D Centre, HLL Lifecare Limited, Thiruvananthapuram, Pincode- 695 017, India; b Dept. of Computational Biology & Bioinformatics, University of Kerala, Thiruvananthapuram, Pincode-695581, India

**Keywords:** Tamoxifene, 5-Hydroxy tamoxifene, Raloxifene, Endoxifene, Ormeloxifene, SERM (Selective estrogen receptor modulator), Cytotoxicity

## Abstract

SERMS like Tamoxifene, 5-hydroxy tamoxifene, raloxifene and endoxifene has been used for the treatment of hormonal imbalances and dependent cancers owing to their action via Estrogen receptors as in the treatment of estrogen sensitive breast cancers. Due to the adverse side effects, modifications and development of the existing or newer SERMS has always been of immense interest. Ormeloxifene, a SERM molecule manufactured by HLL Lifecare Ltd, India as birth control under the trade names Saheli, Novex, and Novex-DS which is also investigated against mastalgia, fibro-adenoma and abnormal uterine bleeding. Anti-cancer effects have been reported in estrogen dependent and independent cancers which shows its wide scope to be implemented in cancer therapy. Current investigation is a comprehensive effort to find the cytotoxic potential of Ormeloxifene in comparison with clinically used four SERMS in twenty six cancer cell lines of different origin using Adriamycin as positive control. Also the computational studies pertaining to selected target/ligand with respect to tumor progression, development, treatment responses and apoptosis. The studies proved effective cytotoxicity of Ormeloxifene on cancer cell lines with lower TGI, GI50 and LC_50_ values which are significantly comparable. Also the *in silico* studies proved that the docking score of the compound suggests the interaction of the compound which could tightly regulate key target genes controlling cancer like ER, EGFR kinase, EGFR-cSRC, HDAC-2, PARP-1 and BRAF. This study brings out the superior efficacy of Ormeloxifene compared to other SERMS with proven safety profile to be repositioned as an anti-cancer drug to treat diverse cancer types.

## Highlights

• Ormeloxifene is a SERM class of compound currently used contraception, treatment of mastalgia, abnormal uterine bleeding and fibro adenoma.
• Our studies prove that Ormeloxifene exerted better cytotoxic effects than other clinically used SERMS
• The computational studies on ten important protein ligands prove that the compound could regulate key pathways in cancer progression.
• The studies put forth efficacy of Ormeloxifene to be repositioned as an anti-cancer drug and the need for more studies.

## Graphical abstract

**Figure:**
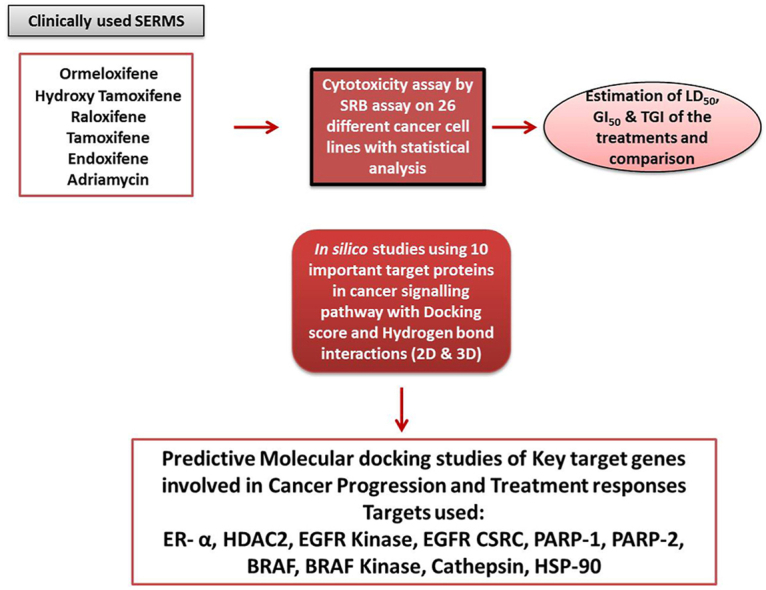

## 1 Introduction

The selective estrogen receptor modulators (SERMs) are a group of drugs which exert selective agonist or antagonist effects on various estrogen target tissues. In estrogens, there are two receptors such as estrogen receptor α and β (ER-α&β). These two receptors are coded by different genes and their tissue expression varies in organs. ER-α is expressed mostly in reproductive tissues, liver and central nerve system (CNS) whereas ER-β is expressed in tissues, bones, urogenital tract, ovaries, prostrate and CNS (Mirecki-Garrido et al., 2012, 2016; Lee et al., 2012). SERMs are currently used in the treatment of various estrogen-related diseases like ovulatory dysfunction for managing infertility prevention and treatment of postmenopausal osteoporosis and treatment for reducing the risk of breast cancer (Tang et al., 2019; Kauffman et al., 2021). Recently, this chemical group of SERMs are being exploited for the development of new target molecules for the treatment of estrogen dependent as well as estrogen independent cancers, abnormal uterine bleeding and other hormone related disorders. In this context, major efforts are needed to develop a new SERM with better therapeutic profile and lesser side effects and cost-effective (Jordan and Brodie, 2007).

The International Agency for Research on Cancer estimates that globally, 1 in 5 people develop cancer during their lifetime, and 1 in 8 men and 1 in 11 women die from the disease. These new estimates suggest that more than 50 million people are living within five years of a past cancer diagnosis. Breast cancer represents 1 in 4 cancers diagnosed among women globally. Colorectal, lung, cervical, and thyroid cancers are also common among women. Lung cancer and prostate cancer are the most common among men, together accounting for nearly one-third of all male cancers (Bray et al., 2018). In this paper, the cytotoxicity of clinically approved SERMS presently used for treatment of estrogenic dependent breast cancer were administered and compared on multiple cancer cell lines of different origin to observe the cytotoxic responses and estrogen independent effects.

Tamoxifen is one of the first generation SERM used as first-line therapeutic drug for all stages of estrogen-dependent breast cancers. It has also been reported to reduce breast cancer incidence in high-risk pre and postmenopausal women. It was found to exert estrogenic effects in other tissues like uterus at the same time (Nabholtz et al., 2000; Kyvernitakis et al., 2018). However the use of tamoxifen could cause resistance after two to five years of therapy, and also threefold increase in incidence of endometrial cancer. The other complications reported include the occurrence of deep vein thrombosis, pulmonary embolism, DNA adducts formation and liver cancer risk (Hemminki et al., 1996). In addition, the occurrence of DNA adducts in leukocyte and endometrial samples from women treated with tamoxifen suggest that it may be genotoxic to humans (Hemminki et al., 1997Hemminki et al., 1997; Shibutani et al. 1998; Beland et al., 1999). The antiestrogen tamoxifen is effective in therapy for breast cancer. However, its use is limited by the eventual development of acquired tamoxifen resistance in many patients. The mechanisms responsible for tamoxifen resistance remain unknown; loss of estrogen receptor (ER), selection of hormone-independent breast cancer clones, or alterations in serum tamoxifen levels after long-term use might be some reasons.

The antagonistic and agonistic properties inherent to TAM are also present in its numerous metabolites, specifically 4′-hydroxy-tamoxifen (4-OH-TAM). With the addition of a hydroxyl group, 4-OH-TAM has been shown to have a higher potency than TAM both *in vitro* and *in vivo* corresponding to a higher affinity for the ER (Lim et al., 2005).

Raloxifene hydrochloride is another SERM, chemically distinct from tamoxifen and estradiol, that binds to estrogen receptors to competitively block estrogen-induced DNA transcription in the breast and endometrium (Sato et al., 1998; Park and Jordan, 2002).Also, a second-generation SERM, approved by Food and Drug Administration (FDA) to decrease the risk of invasive breast cancer in postmenopausal women is reported to be a teratogenic drug (Adomaityte et al., 2008). Both tamoxifen and raloxifene increase hot flashes and, therefore, may be best tolerated by women who are no longer having hot flashes after menopause. Raloxifene is reported to induce deep vein thrombosis (DVT) risk and pulmonary edema (Jacobs et al., 1999). Endoxifen is a major active metabolite of tamoxifen that is being investigated for clinical use.

Endoxifen is known to elicit greater anti-estrogenic effects in breast cancer models compared with its parent compound tamoxifen (Lim et al., 2006; Wu et al., 2011) and in tamoxifen-treated breast cancer patients, endoxifen concentrations were reported to positively associated with disease-free survival (Moyer et al., 2011; Thoren et al. 2021). For these reasons, endoxifen is currently being investigated as a novel endocrine therapy for the treatment of estrogen receptor (ER)α–positive breast cancers and phase I and II clinical trials (NCT02311933, NCT01327781, and NCT01273168) have been done.

Ormeloxifene (1-[2-[4-[(3R, 4S)-7-Methoxy-2, 2-dimethyl-3-phenyl-chroman-4yl]phenoxy]ethyl]pyrrolidine. Hydrochloride) an orally active non-steroidal and non-hormonal contraceptive agent developed by the Central Drug Research Institute, Lucknow, India and marketed by HLL Lifecare limited, India under the brand name of Saheli, Novex-DS (60 ​mg), and Novex (30 ​mg) may be a better alternative against these cancer therapies (Lal et al., 1995; Kaushik et al., 2018). Drug repositioning is considered as a cost-effective mode for drug development and circumvents safety concerns of FDA-approved known drugs for other novel indications (Pillaiyar et al., 2020). Ormeloxifene was tested for its therapeutic efficacy against various cancer cell line models and *in vivo* animal model for breast cancer. Ormeloxifene is now at early stages of clinical development for the treatment of breast cancer, Osteoporosis and mastalgia **(**Hafiz et al., 2018). In one clinical trial, breast cancer patients were treated with ormeloxifene (60 ​mg, three times a week) for 4–6 weeks. It was reported that about 38.5% of breast cancer patients responded to the ormeloxifene therapy and older postmenopausal females patients showed relatively better anticancer activity. The responses to ormeloxifene treatment were more promising for bone, pulmonary, soft tissue, skin, and lymph-node metastases than for visceral metastases. However, there was no correlation between the number of lesions or estrogen receptor positivity and response to ormeloxifene therapy (Pillai et al., 2018). Ormeloxifene is a potent agent that has been widely shown to act upon several important molecular targets in cancer *in-vitro*. Another reason for effectiveness against cancer treatment is that this molecule possess excellent therapeutics index with no systemic toxicity even at chronic administration. Studies indicated that ormeloxifene has potential to treat both ER positive and negative breast cancer and provide a strong basis for repurposing its use from current usage in contraception and treatment of abnormal uterine bleeding to chemotherapeutics (Agrawal et al., 2016). Also the wide clinical scope of this SERM as a therapeutic intervention for a broad range of disease conditions is yet to be intensively explored. Major efforts are needed to develop a new non-hormonal SERM with better therapeutic profile and lesser side effects. This study comprehensively investigates the scope of potential of Ormeloxifene to be developed as a better effective and prospective SERM, in comparison with other SERMS against ER dependent as well as ER independent cancers by *in-vitro* cytotoxicity assay on twenty six different cancer cell lines of different origin and predictive molecular docking studies on various important target genes involved in cancer progression and treatment responses. Molecular Docking was used in this study to position the computer-generated 3D structure of small ligands into a receptor structure in a variety of orientations, conformations and positions which could be useful in providing insights into molecular recognition.

## 2 Materials and methods

### 2.1 Chemicals and consumables

RPMI-1640 Medium, DMEM medium and Fetal Bovine Serum (FBS) were purchased from Invitrogen, USA. Trypsin was purchased from Sigma-Aldrich, USA. Adriamycin was purchased from Pfizer Ltd, Italy. Plastic wares were purchased from Eppendorf, Germany. All the other chemicals and consumables required for the study were procured from local suppliers throughout the experimentation.

### 2.2 Cell culture

The twenty-six cancer cell lines used for this study were procured from National Cancer Institute (NCI), USA and National Centre for Cell sciences (NCCS), Pune and maintained as per standard testing guidelines. Cancer cell lines from diverse tissue origin (breast, cervix, ovary, prostate, liver, colon, kidney, lung, brain, oral, bladder, blood, pancreas, and skin) were employed for the drug screening assay to study and compare the efficacy of the selected SERMS (Table 1). The sub-clones used for the experiment include Breast cancer cell lines: T47D:C4:SW, MDA-MB-435/β4, MCF-7A3, Cervical cancer cell lines: ME-180/TNF, HeLa–S3-5, Ovarian cancer cell lines: SKOV3-PM2, A2780/E6, Prostate cancer cell lines: DU-145-C1-I, PC3-M1, Leukemia: U937 PLUS, JURKAT E6, HL-60/MX1, Hepatic cancer cell lines: HePG2-C12E1-43, Pancreatic cancer cell line: MIA-BCL2, Bladder: T24M, Kidney: Vero E6, HEK 293-G, Keratinocyte: Haca T ras.

**Table 1:** Different cell lines and its tissue of origin.

Sl. No	Cell line	Tissue of origin	Sl. No	Cell line	Tissue of origin
**1**	MCF7	Breast	**14**	Jurkat	Leukemia
**2**	MDA-MB-435	Breast	**15**	HL-60	Leukemia
**3**	T47D	Breast	**16**	RPMI-8226	Myeloma
**4**	ME-180	Cervix	**17**	U937	Lymphoma
**5**	HeLa	Cervix	**18**	SKMEL-2	Melanoma
**6**	A2780	Ovary	**19**	Haca T	Keratinocyte
**7**	SK-OV-3	Ovary	**20**	T-24	Bladder
**8**	DU145	Prostate	**21**	NCIH226	Lung
**9**	PC- 3	Prostate	**22**	MIA-PA-CA2	Pancreas
**10**	PLC-PRF-5	Hepatoma	**23**	SCC-29B	Head & neck
**11**	Hep G2	Hepatoma	**24**	HEK-293	Kidney
**12**	Vero	kidney	**25**	SCC-40	Esophageal
**13**	COLO-205	Colon	**26**	U-373MG	Glioma

### 2.3 Cytotoxicity assay using sulforhodamine B (SRB)

Based on Skehan P et al. (1990) the sulforhodamine B (SRB) assay is a rapid, sensitive and inexpensive method used for the quantification of cellular proteins and was subsequently adopted by the National Cancer Institute for *in-vitro* anti-tumor screening (Skehan et al., 1990; Vichai and Kirtikara, 2006).This method provides a sensitive measurement of drug-induced cytotoxicity and its active concentration ranges. In the present study, the cell lines were grown in RPMI 1640 or DMEM medium containing 10% FBS supplemented with antibiotic solution and 2 ​mM L-glutamine. For the screening experiment, cell lines in their 24th to 35th passage were taken. Jurkat,U-373MG, HEK-293, U937 and RPMI-8226 (24th passage), HL-60, SKMEL-2, ME-180, DU145, HeLa and SCC-29B (25th passage), Haca T, T47D, Hep G2 (26th passage), COLO-205, MCF7, SCC-40, NCIH226 (27th passage), PLC-PRF-5, A2780 and T-24 (29th passage), MIA-PA-CA2 and PC- 3 (31st passage) and MDA-MB-435, SK-OV-3 and Vero (35th passage). The mycoplasma contamination of the cell line was ruled out by Hoechst staining in the previous passages. Mycoplasma negative cultures were only used for the experiments. Cells were inoculated into 96-well microtiter plates in 100 ​μL ​at plating densities depending upon the doubling time of individual cell lines. After cell inoculation, the microtiter plates were incubated at 37 ​°C, 5% CO_2_, 95% humidified air for 24 ​h prior to addition of experimental drugs. Different SERMS namely, Ormeloxifene HCL, Hydroxy Tamoxifene, Raloxifene, Tamoxifene, Endoxifene and Adriamycin (Positive control) were initially solubilized in Dimethyl sulfoxide (DMSO) and added to cells at final concentrations of 10, 20, 40 and 80 ​μg/ml and plates were incubated for 48 ​h. Each experiment was performed in triplicates. To record morphological changes in cell cultures after 48-hrs of incubation, cells treated at highest drug concentration were imaged using Phase Contrast Inverted Microscope (Model Eclipse Ti–S, NIKON Co., Japan) fitted with digital camera to the computer. Further, the cells were fixed using 10% or 16% Tricholoro acetic acid for adherent and non-adherent cultures, respectively. Then cells were stained with SRB dye and bound stain in the cells was subsequently eluted with 10 ​mM Trizma base. The absorbance was read on a plate reader (Model Sunrise, Tecan Inc., USA), at a wavelength of 540 ​nm with 690 ​nm reference wavelength. Percentage growth was calculated on a plate by plate basis for test wells relative to control wells. Percentage growth was expressed as the ratio of average absorbance of the test well to the average absorbance of the control wells.

Percentage of control cell growth ​= ​Mean OD sample- Mean OD day 0 X 100

Mean OD negative control- Mean OD day 0

Percentage growth inhibition ​= ​100 - % of control cell growth

It is possible to use the SRB assay to determine the LD_50_ values of compounds from the dose response relationship between the compound concentration and the percentage of cells killed, which is calculated using the formula below.

Percentage of cells killed ​= ​100 - Mean OD sample X 100

Mean OD day 0

The following values which implicates the cytotoxicity of each drug on each cell line namely LD_50_ (Concentration of drug causing 50% cell kill), GI_50_ (Concentration of drug causing 50% inhibition of cell growth) and TGI (Concentration of drug causing total inhibition of cell growth) were calculated. GI_50_ value of ≤10 ​μg/ml is considered to demonstrate good cytotoxic activity.

### 2.4 Statistical analysis

Data were expressed as mean of independent experiments in the *in vitro* cytotoxicity experiments. The data was analyzed by the mean graph technique to investigate the individual sensitivity to different SERMS of each cancer cell line. In this method the most active and less toxic SERMS were observed. For the *in vivo* experiments, results were expressed as Mean ​± ​Standard deviations. Statistical analysis were done using one way Anova followed by Tukey multiple comparison tests using SPSS. P value ​< ​0.05 was considered statistically significant.

### *2.5* *In-silico* studies

*In silico* studies were done using the protocols and methods obtained from the references (Pang et al., 2018; Hung et al., 2014; Nasab et al., 2018; Chen et al., 2020; Abbasi-Radmoghaddam et al., 2021; Sherstyuk et al., 2020; Luo et al., 2008; Dong et al., 2013; Nastasa et al. 2019; Nurhayati et al., 2015). The molecular mechanism of the SERM class of compounds such as Bazedoxifene, Raloxefene, Tamoxifene and Ormeloxifene were compared against selective drug targets with Adriamycin and standard drug for each target by molecular docking studies. The computational approach helps to understand the binding affinity and interaction of the drugs with critical amino acid residues of the target proteins. The preprocessing step of molecular docking included preparation of both target proteins and ligands. The crystal structure of each target protein was retrieved from Protein Data Bank. The protein structures were preprocessed by removing the bounded ligands and convert into a most minimized and energy stable conformation. The active sites for docking was selected based on inhibitor binding site and also from receptor cavities of the protein. The drug models were retrieved from Pub Chem and preprocessed by generating its conformers and converted into most minimized structures for docking. The molecular properties of the ligands like number of Hydrogen bonding donor, number of hydrogen bonding acceptor, A Log P value, molecular weight (Daltons) and toxicity were screened under Lipinski's rule of five, to validate whether they are druggable compounds or not and toxicity of the ligands were calculated. The octanol**/**water partition coefficient is defined as the ratio of a chemical's concentration in the octanol phase to its concentration in the aqueous phase of a two-phase octanol/water system and hence A Log P value does not have a specific unit. Molecular docking of each target with Bazedoxifene, Adriamycin, Raloxefene, Tamoxifene, Ormeloxifene and standard drugs was performed by Lib dock module of Discovery studio. The binding site of proteins were selected based on the antagonistic action of drugs. After docking, the bound protein ligand complex were analyzed by considering the active site amino acids bounded with drug molecule. Docking score, number of hydrogen bonds made by the ligands with target active site amino acid residues and bond distance were calculated. The target proteins with its PDBIDs and the criteria for selection of target proteins based on its importance in cancer studies are listed below with references.

**Estrogen receptor alpha (3ERT)** a transcription factor that regulates gene expression events that culminate in cell division and contributes to its critical role in mammary gland development (Allred et al., 2004) ER alpha promotes breast cancer initiation and proliferation as well as oncogenic protein expression, such as Cyclin D1 and c-Myc, while it inhibits the level of cell cycle inhibitors, including P21 (Wong et al., 2001; Cariou et al., 2000).

**HDAC 2 (5IWG)** Overexpression of HDAC2 is an indicator of poor prognosis of breast cancer patients who have elevated expression of a multidrug resistance-associated protein. Targeted inactivation of HDAC2 is observed to restore p16INK4a activity and exerts antitumor effects on human gastric cancer (Kim et al., 2013). HDAC2 was reported to confer oncogenic potential to human lung cancer cells by deregulating expression of apoptosis and cell cycle proteins (Jung et al., 2012).

**EGFR kinase (5UGA),** EGFR is found on the surface of some normal cells that is involved in cell growth and belongs to the receptor tyrosine kinases. Blocking EGFR may keep cancer cells from growing (Dawood et al., 2019; Methot et al., 2008).

**EGFR C-Ssrc (4MXO),** c-Src phosphorylates specific tyrosine residues in other tyrosine kinases (Wheeler et al., 2009). It plays a role in the regulation of embryonic development and cell growth. An elevated level of activity of c-Src is suggested to be linked to cancer progression by promoting other signals. Src, as a mediator of receptor transactivation, can uniquely activate EGFR in the absence of EGFR ligand, and a Src inhibitor is synergistic with an EGFR monoclonal antibody *in vitro* in eliciting growth inhibition. Src inhibition is also reported to be acting in a synergistic manner in *in vivo* experiments treated with platinum chemotherapeutics, further increasing the potential of combination regimens with Src inhibitors.

**PARP1(5WS1),** the most abundant isoform of the PARP superfamily, is a chromatin-associated protein and plays a significant role in cell proliferation, malignant transformation, transcriptional regulation, apoptosis and DNA repair mechanisms (Amé et al., 2004). PARP 1 is reported to induce cell survival through DNA repair by cleaving into two fragments by activated caspases resulting in its inactivation during apoptosis (Ossovskaya et al., 2010). Overexpression of PARP1 is found in different primary human tumours compared to normal tissue counterparts (Germain et al., 1999; Rojo et al., 2012).

**PARP2 (3KJD)** mediates glutamate and aspartate ADP-ribosylation of target proteins: the ADP-D-ribosyl group of NAD^+^ is transferred to the acceptor carboxyl group of glutamate and aspartate residues and further ADP-ribosyl groups are transferred to the 2′-position of the terminal adenosine moiety, building up a polymer with an average chain length of 20–30 units (Vyas et al., 2014).

**BRAF (2FB8)** is a Serine-Threonine protein kinase that belongs to the highly oncogenic RAS/RAF/MEK/ERK signalling pathway (Lavoie and Therrien, 2015).The studies pertaining to the regulation of BRAF gene expression could contribute to a deeper understanding of the functioning and deregulation of the gene for targeted therapy.

**Cathepsin D (4OD9**), gene has been reported to act as both housekeeping gene and as a hormone-regulated gene (Cavailles et al., 1993) Overexpression of cathepsin D is reported to facilitate breast cancer metastasis. Procathepsin-D, a premature cathepsin-D form was reported to be abundant in breast cancer, having autocrine properties, inducing cell proliferation in MCF-7 breast cancer cells (Vignon et al., 1986).

**Fig. 1:**
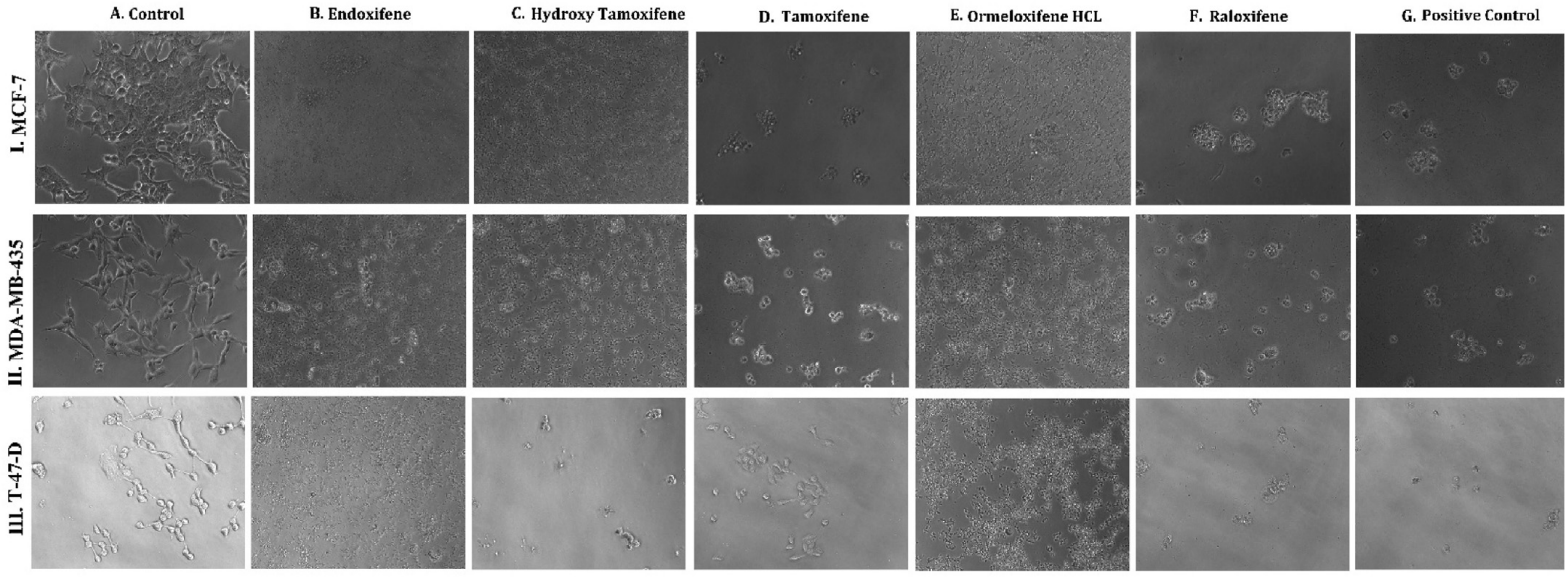

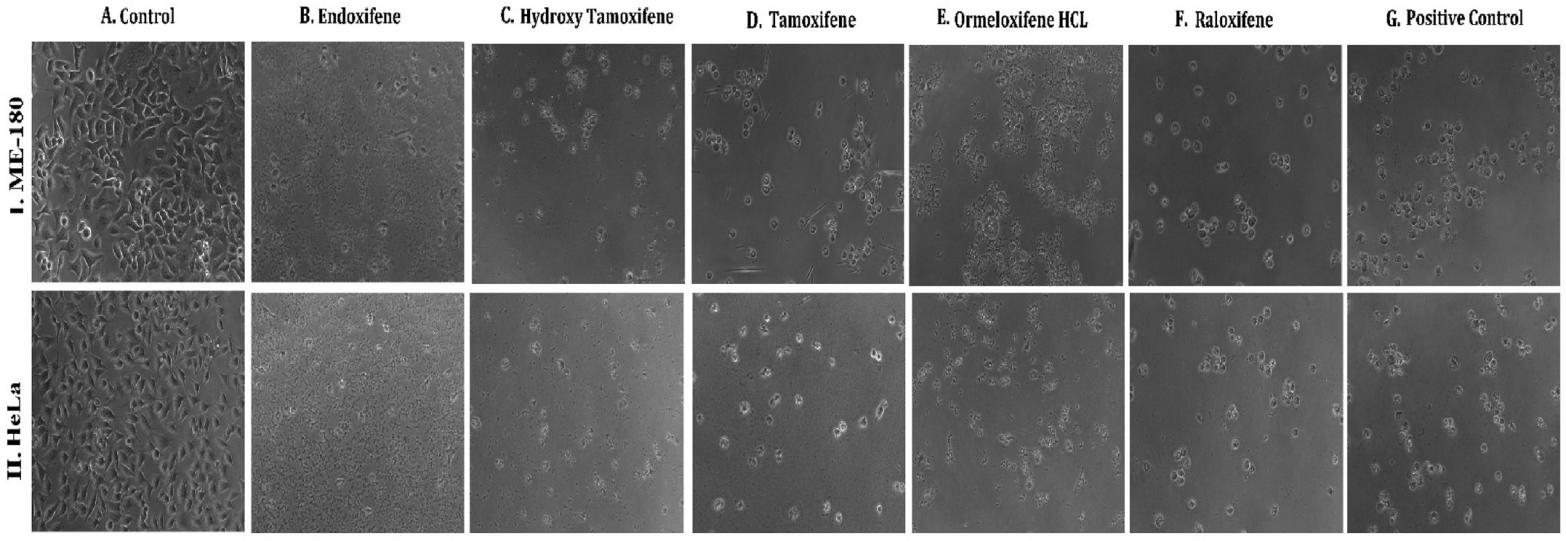

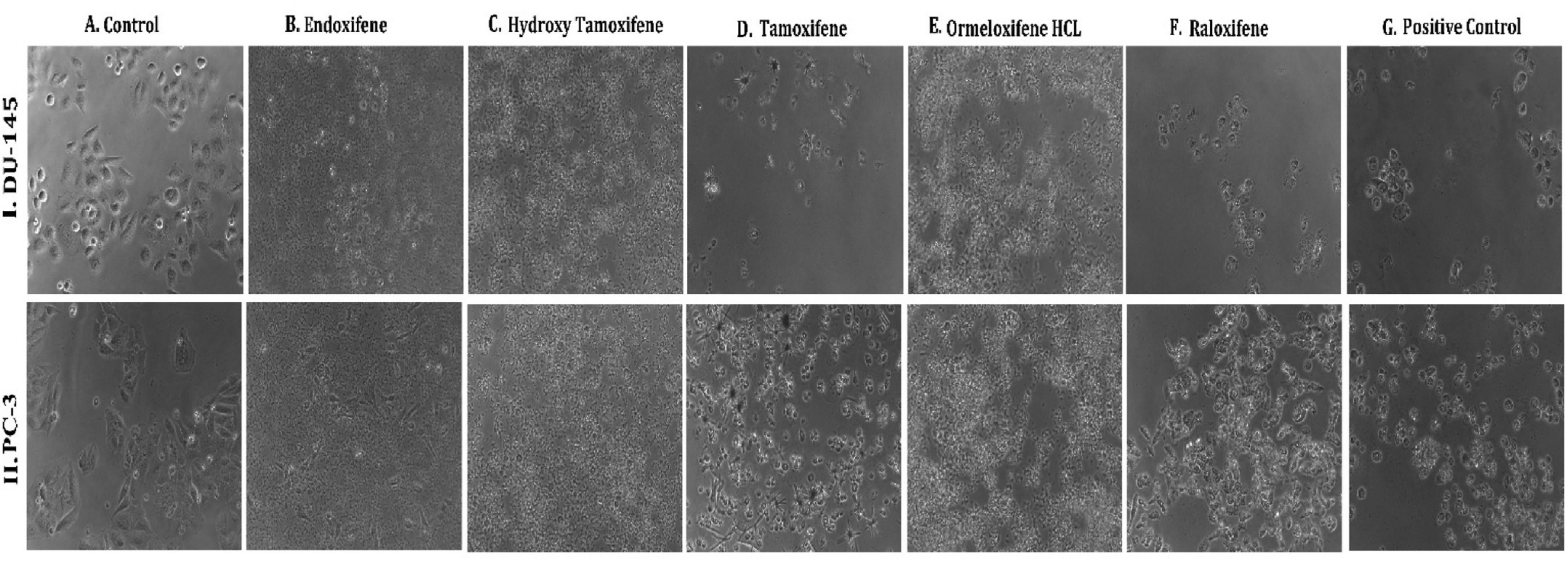

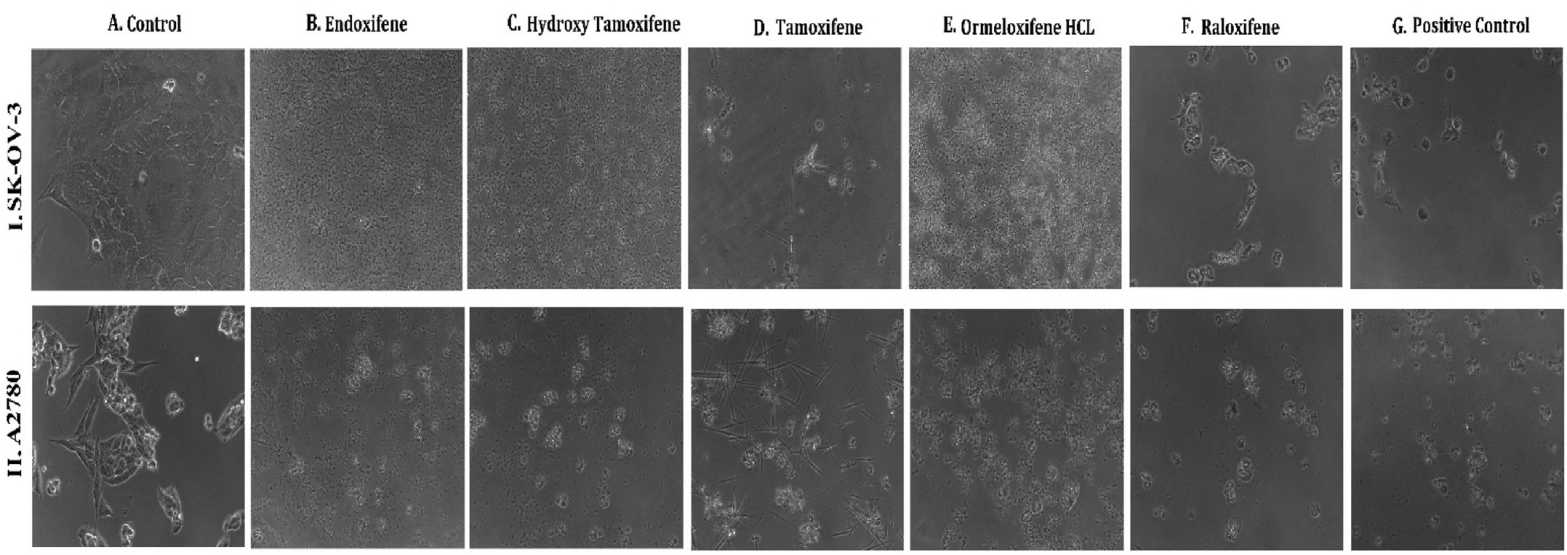

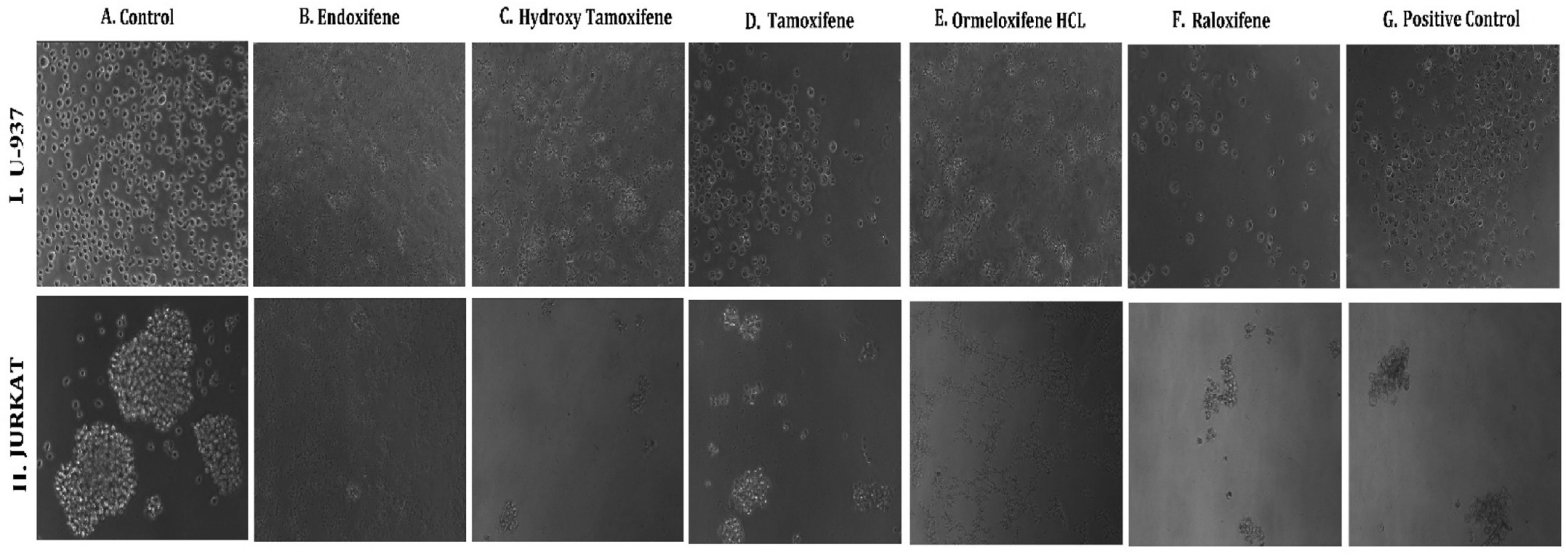

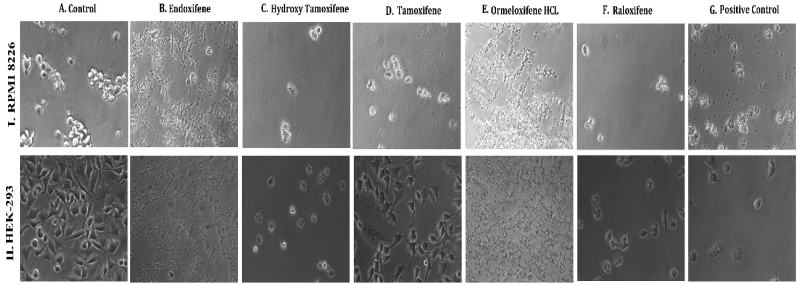
**Phase contrast microscopic images of cells showing the morphological differences in control Vs treated cells on different cell lines:** (I.MCF-7, II. MDAMB-231, III. T-47D) cancer cell lines (A) Untreated Control cells, Cells treated with (B) Endoxifene, (C) Hydroxy tamoxifene), (D) Tamoxifene, (E) Ormeloxifene HCL, (F) Raloxifene, (G) Adriamycin treated positive control.

**HSP 90 (1UY8) HSP-90** over expression has been reported to exert resistance in cancer cells by evolving cells to become resistant to various stimuli and stress. So pharmacological inhibition of HSP 90 could provide therapeutic interventions in cancer treatment (Zagouri et al., 2012).(see Fig. 1).

## 3 Results and discussions

### *3.1* *In vitro* cytotoxicity assay

**Fig 2:**
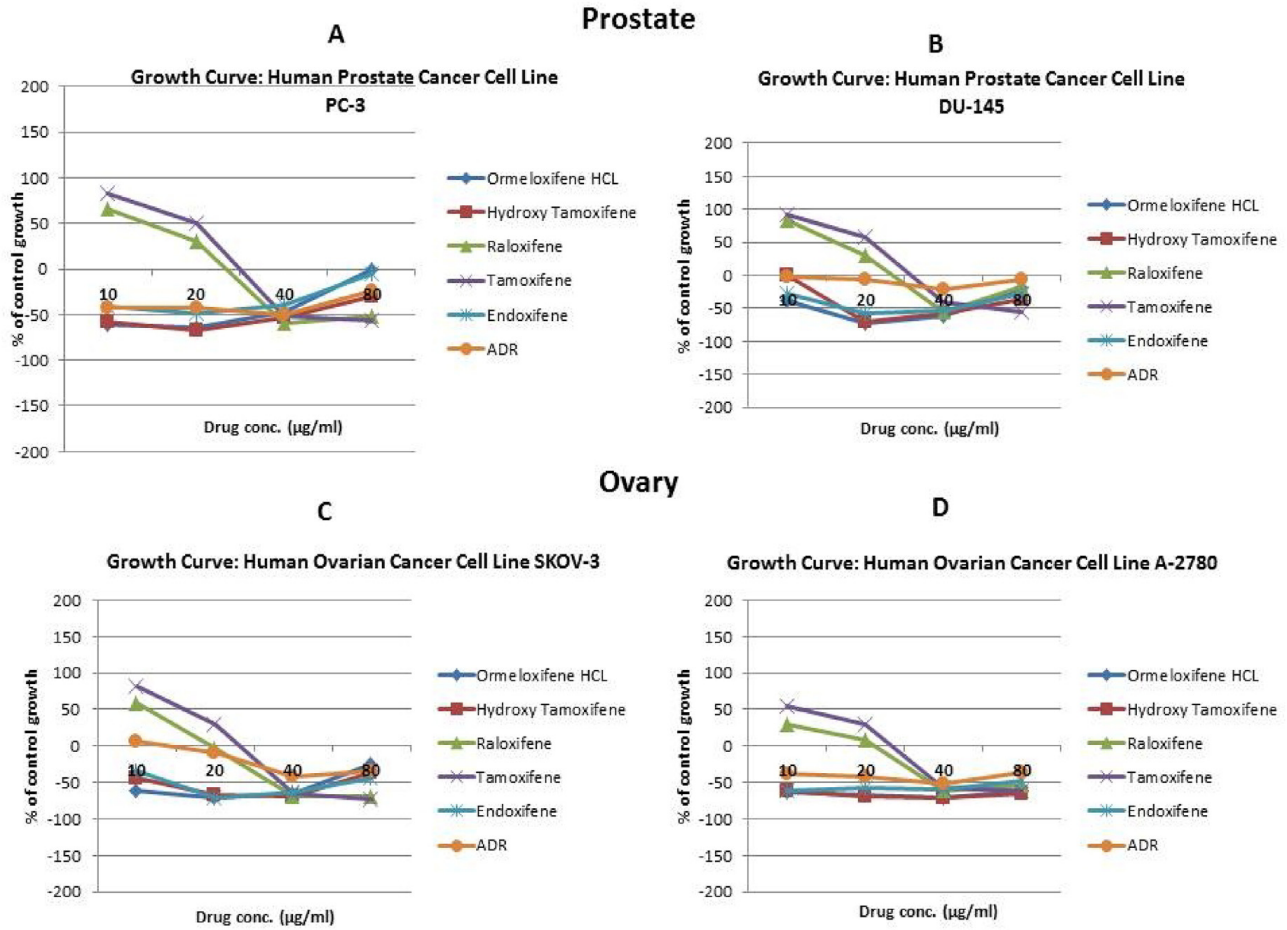

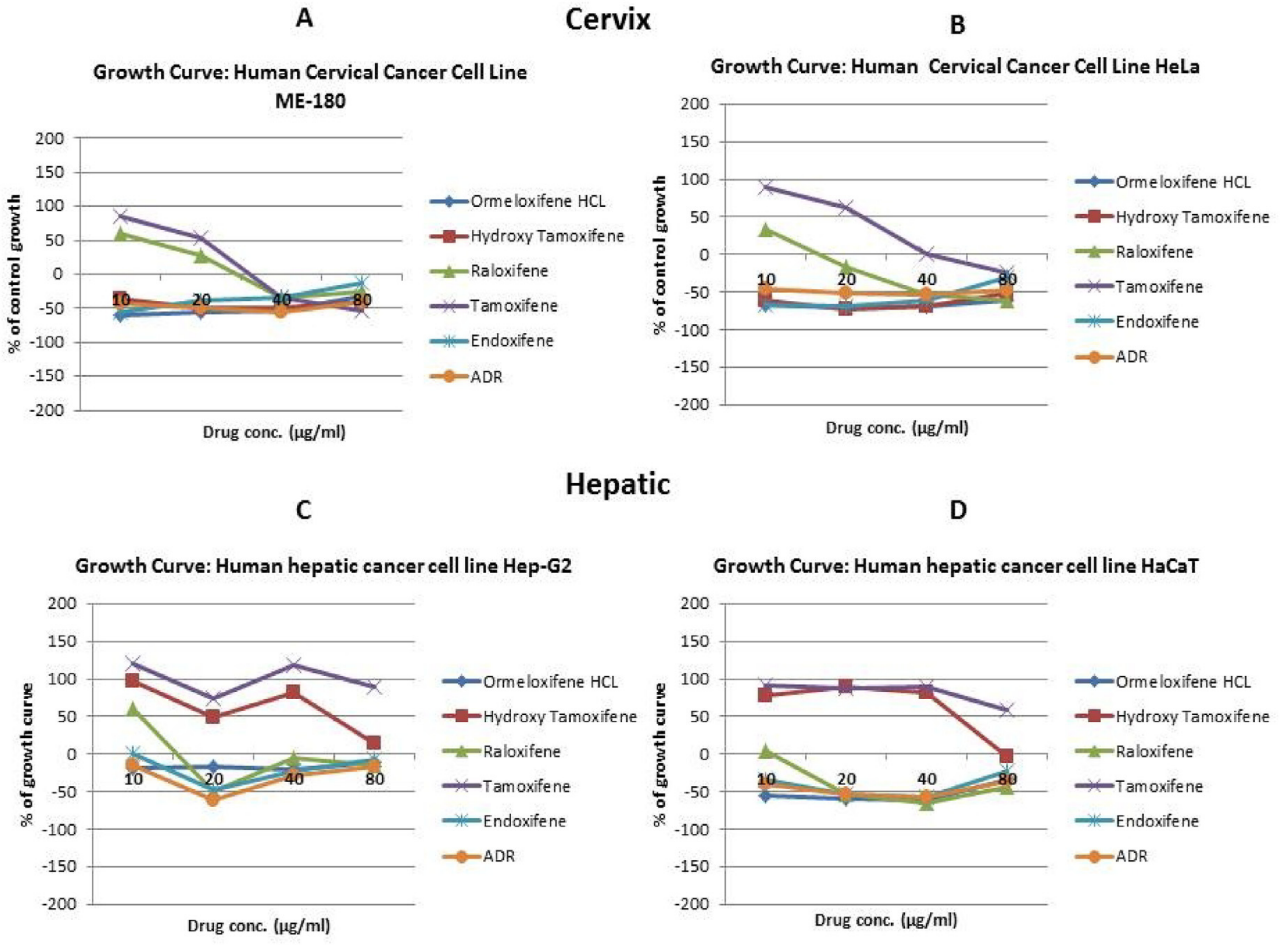

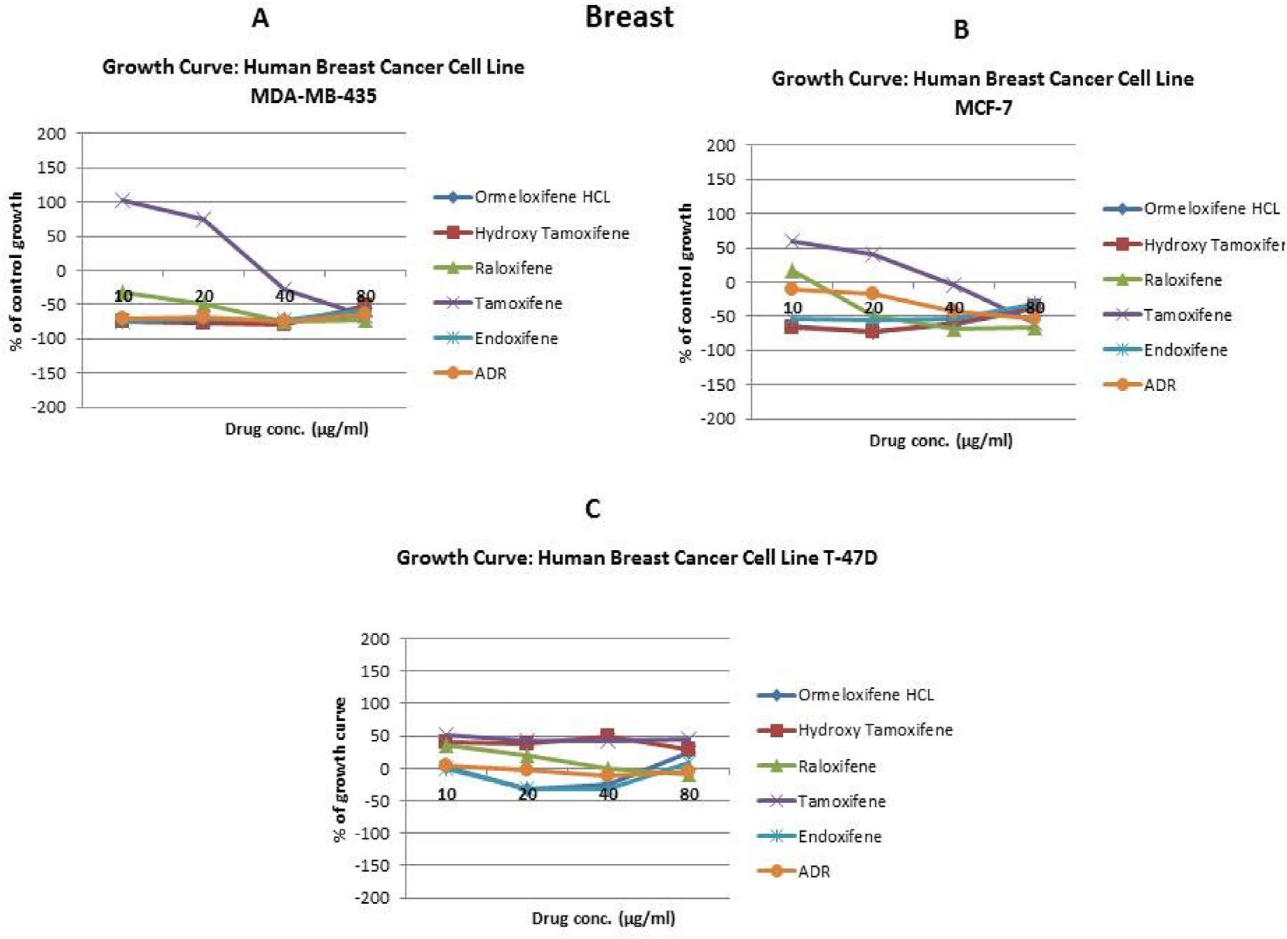

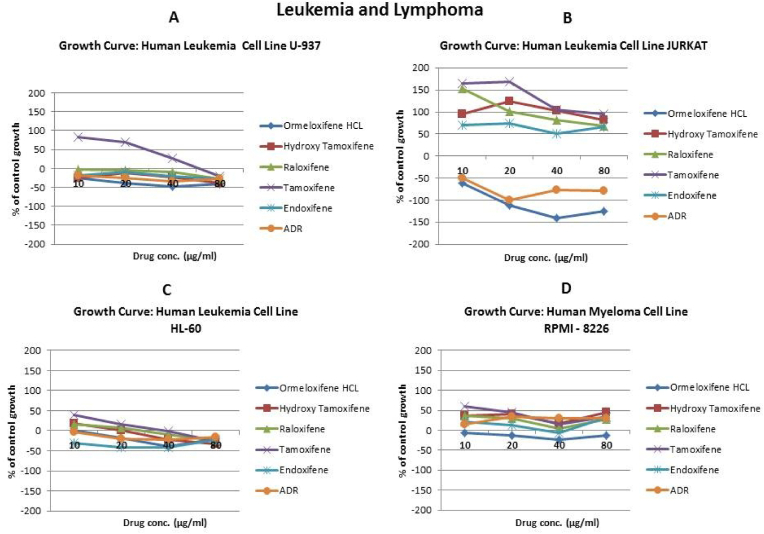

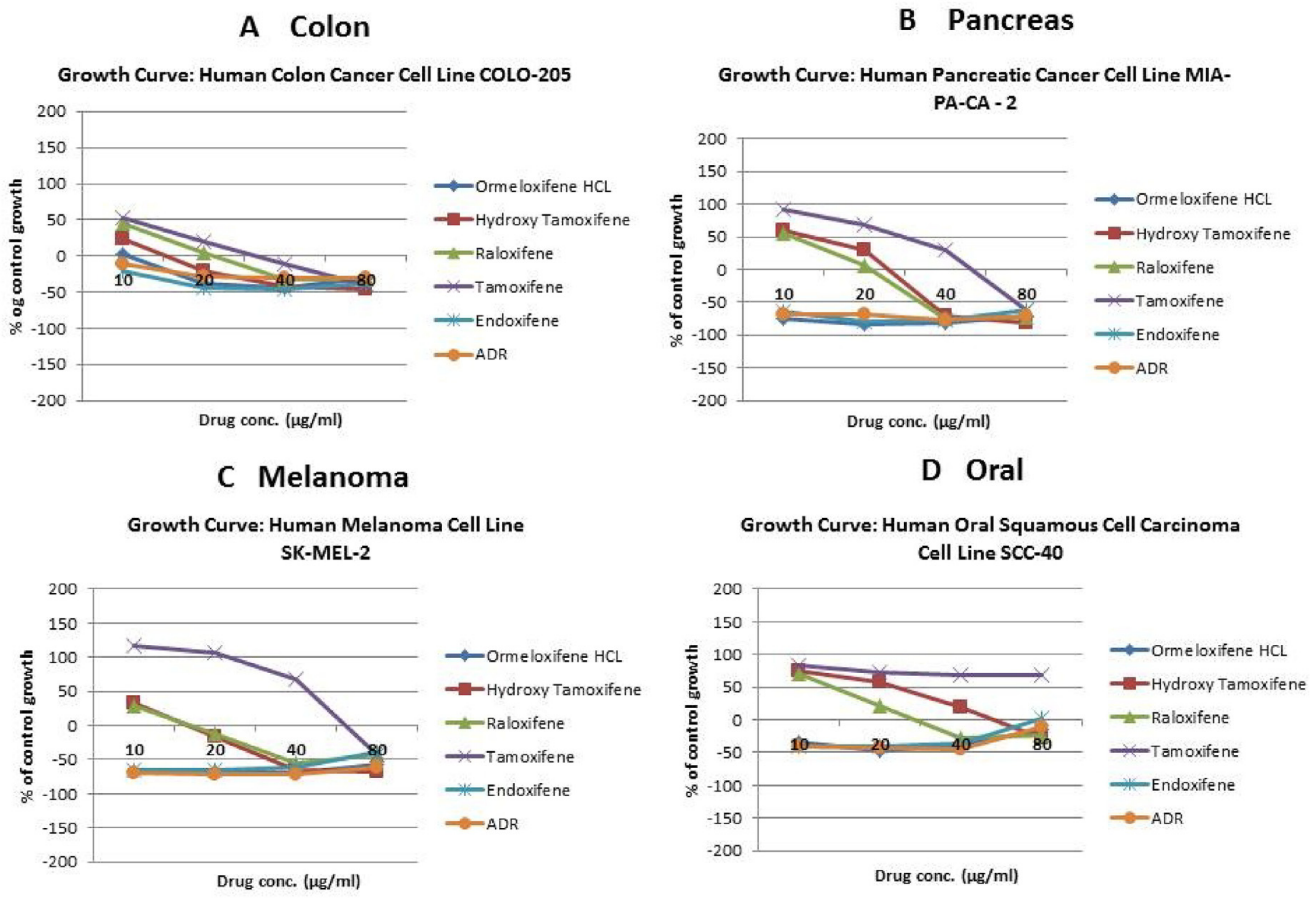

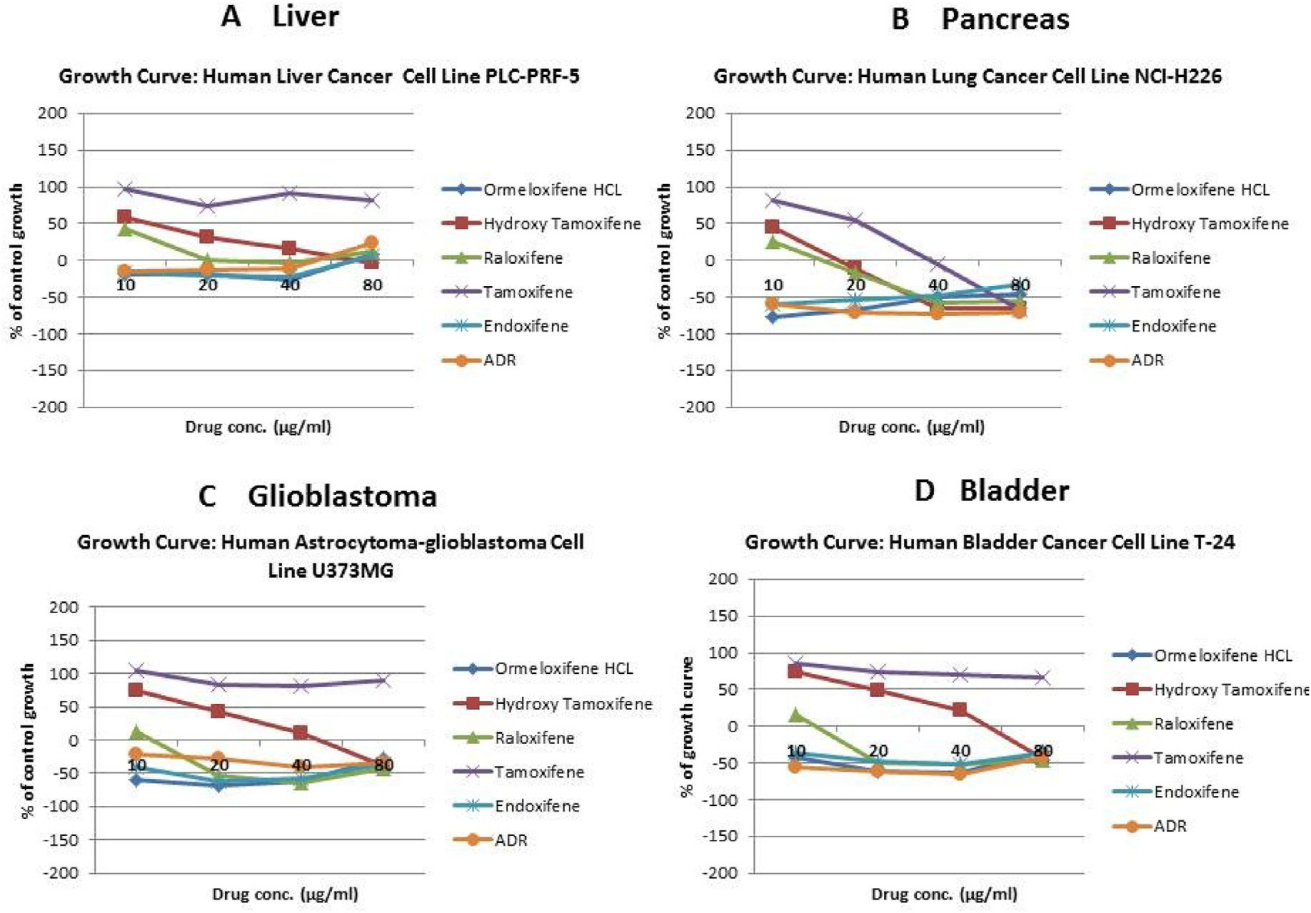

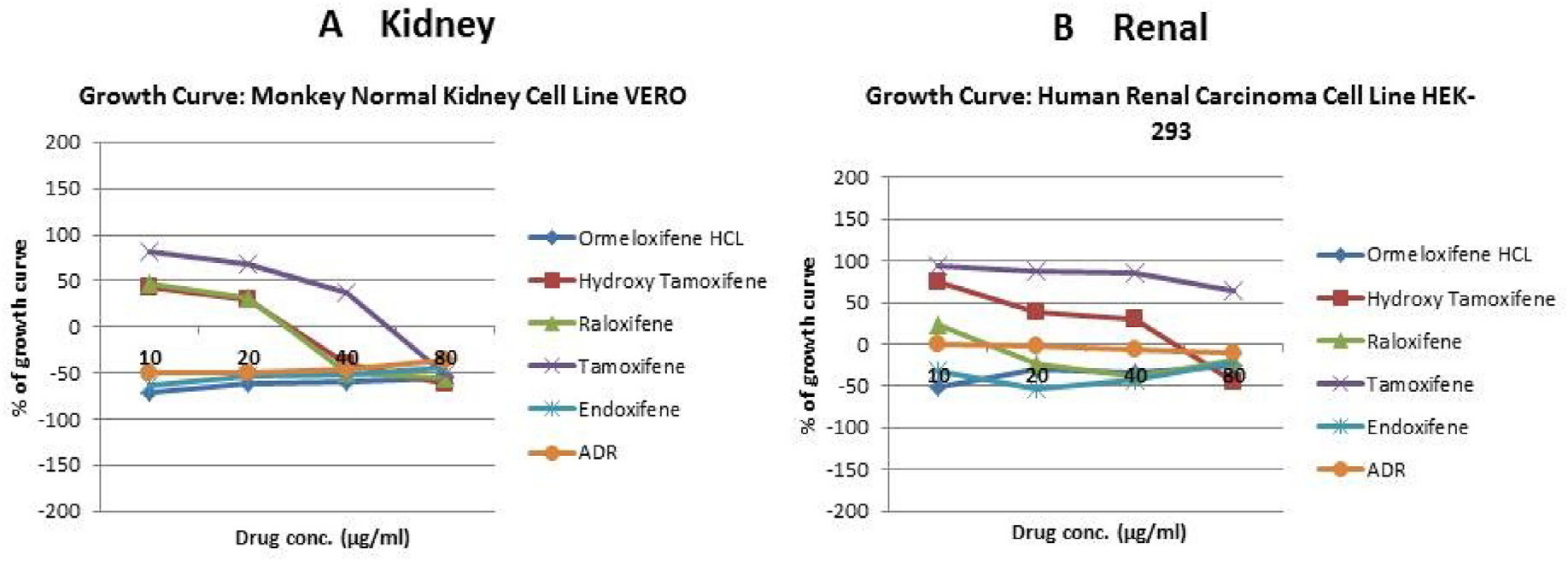
Prostate (PC-3, DU-145) and ovarian (SK-OV-3, A-2780) cancer cell lines (A) PC-3 (B) DU-145 (C) SK-OV-3 (D) A-2780 cell lines treated with 10,20,40 and 80 μg/ml of ormeloxifene, hydroxy tamoxifene, raloxifene, tamoxifene, enoxifene and adriamycin.

In order to evaluate *in vitro* cytotoxic activity of Ormeloxifene in comparison with related SERMS, Hydroxy-tamoxifene, raloxifene, tamoxifene and endoxifene, these compounds were tested against a panel of 26 cancer cell lines of 17 different tissue origins (Table 1). The *in-vitro* testing was conducted using four concentrations 10, 20, 40 and 80 ​μg/ml. Phase contrast microscopy was done in the following cancer cell lines (Breast-MCF-7, MDAMB-231, T47D, Cervical-ME-180, HeLa, Ovarian-A-2781, SK-OV-3, Prostate-PC-3- DU-145, Leukemia-Jurkat, Myeloma-RPMI-8226, Renal-HEK-293, Glioblastoma-U937). The microscopic imaging of the cultures at highest tested concentrations (40 ​μg/ml) demonstrated cell shrinkage, membrane blebbing, cell fragmentation and detachment from substratum (Fig. 1(A-F). There was also gross decrease in cell numbers with increase in concentration of SERMS. Untreated cell control exhibited intact morphology of cells while adriamycin-treated positive control cultures showed maximum efficacy on cells. represents the line graphs for dose response of the compounds against various cancer cell lines, Prostate and ovarian cancer cell lines (Fig. 2A), Cervical and Hepatic cancer cell lines (Fig. 2B), Breast cancer cell lines (Fig. 2C), Leukemia and Myeloma cell lines (Fig. 2D), Colon, Pancreatic, oral and Melanoma cancer cell lines (Fig. 2E), Liver, Lung, Glioblastoma and bladder cancer cell lines (Fig. 2F), Kidney and Renal cancer cell lines (Fig. 2G). Using these graphs, the chemo sensitivity response parameters GI_50_ (50% growth inhibition), TGI (total growth inhibition) and LC_50_ (50% lethal concentration) were extrapolated (Table 2). Cell control was considered as 100% cell growth, Adriyamycin was considered as positive control and exhibited lytic effect on cells seeded i.e. −50% cell growth. As per NCI guidelines, GI_50_ value of ≤10 ​μg/ml was considered to demonstrate good inhibitory activity in case of pure compounds. The GI_50_ value for compounds tested were found to be less than 10 ​μg/ml in Ormeloxifene treated cancer cells which was comparable to that shown by clinically used endoxifene, and raloxifene and standard anti-cancer drug adriyamycin, as illustrated in Table 2. Whereas the compounds tamoxifene and hydroxy-tamoxifene exhibited variable response and also reduced cytotoxicity as evident from the GI_50_ value range in the various cancer cell lines tested. The GI_50_ ​< ​10 ​μg/ml in all cell lines whereas TGI<10 ​μg/ml except in T47d (52.9 ​μg/ml),>80 ​μg/ml in HepG2 and Non-effective (NE) in Jurkat cells. In Adriamycin treated cells, GI5<10 ​μg/ml in all cell lines and TGI was non-effective in A2780, DU-145, HepG2, Jurkat and RPMI-8226 ​cell lines. Tamoxifene was active only against five cell lines viz. T-47D, A2780, Colo-205, HL-60 and RPMI-8226, For the rest eleven cell lines, GI_50_ values of tamoxifene ranged from 13.1 to 30.6 ​μg/ml while for remaining 10 ​cell lines tamoxifene was ineffective and GI_50_ values were >80 ​μg/ml. In Hydroxy tamoxifene treated cells, GI_50_ ​< ​10 ​μg/ml in all cell lines except HepG2 (56.4 ​μg/ml), Jurkat (>80 ​μg/ml), HacaT (46.6 ​μg/ml), T24 (22.5 ​μg/ml), SCC-29B (17.8 ​μg/ml), HEK-293 (21.4 ​μg/ml), SSC-40 (24.8 ​μg/ml) and U37MG (19.4 ​μg/ml). It was evident that Ormeloxifene showed significant cytotoxic effectiveness against all cancer cell lines tested (GI_50_ ​≤ ​10 ​μg/ml) compared to tamoxifene and hydroxytamoxifene. Also it was interesting to note that Ormeloxifene exhibited significantly increased cytotoxicity than that exhibited by standard drug adriyamycin in the cell lines MCF-7, ME-180, A2780, SK-OV3, PC-3, DU-145, HEK-293 and U373-MG (see Fig. 2).

**Table 2:** Chemosensitivity response parameters GI_50_ (50% growth inhibition), TGI (total growth inhibition) and LC_50_ (50% lethal concentration of different SERMS in different cancer cells.

Cell lines	Ormeloxifene HCL			Hydroxy Tamoxifene			Raloxifene			Tamoxifene				Endoxifene				Adriamycin		
	LC50	TGI	G150∗	LC50	TGI	G150∗	LC50	TGI	G150∗	LC50	TGI	G150∗	LC50		TGI	G150∗	LC50		TGI	G150∗
MCF-7	<10	<10	<10	<10	<10	<10	46.8	<10	<10	71.8	42.5	13.1	<10		<10	<10	<10		<10	<10
MDA-MB-435	<10	<10	<10	<10	<10	<10	23.2	<10	<10	66.4	45.8	25.3	<10		<10	<10	<10		<10	<10
T47D	NE	52.9	<10	NE	NE	<10	NE	55.7	<10	NE	NE	<10	NE		NE	<10	NE		<10	<10
ME-180	39.3	<10	<10	NE	<10	<10	>80	42.9	<10	69.6	44.0	18.4	10.1		NE	<10	<10		<10	<10
HeLa	<10	<10	<10	<10	<10	<10	58.9	16.3	<10	88.2	57.0	25.9	<10		<10	<10	<10		<10	<10
A-2780	NE	<10	<10	NE	<10	<10	63.6	20.8	<10	62.1	32.2	<10	77.0		<10	<10	NE		NE	<10
SK-OV-3	46.2	<10	<10	NE	<10	<10	55.1	24.4	<10	58.1	34.4	10.6	NE		NE	<10	>80		<10	<10
DU-145	<10	<10	<10	NE	<10	<10	>SO	45.3	6.5	68.1	43.9	19.8	NE		<10	<10	NE		NE	<10
PC-3	<10	<10	<10	<10	<10	<10	66.0	35.2	<10	66.0	40.8	15.7	<10		<10	<10	<10		<10	<10
PLC-PRF-5	<10	<10	<10	NE	70.6	<10	>80	20.0	<10	>80	>80	>80	>80		<10	<10	>80		<10	<10
Hep G2	NE	>80	<10	NE	>80	56.4	NE	56.2	<10	NE	>80	>80	NE		NE	<10	NE		NE	<10
VERO	<10	<10	<10	65.1	32.6	<10	66.0	32.9	<10	>80	54.9	28.9	<10		<10	<10	<10		<10	<10
COLO-205	>80	<10	<10	72.4	10.6	<10	85.8	33.4	<10	82.4	41.7	<10	>80		<10	<10	>80		<10	<10
JURKAT	NE	NE	<10	>80	>80	>80	>80	>80	>80	>80	>80	>80	>80		>80	>80	NE		NE	<10
HL-60	>80	<10	<10	>80	24.1	<10	>80	32.9	<10	>80	45.4	<10	>80		<10	<10	>80		<10	<10
RPM1- 8226	NE	<10	<10	NE	NE	<10	NE	NE	<10	NE	NE	<10	NE		NE	<10	NE		NE	<10
U-937	NE	<10	<10	NE	<10	<10	NE	<10	<10	>80	63.9	30.6	NE		<10	<10	NE		<10	<10
SK-MEL-2	<10	<10	<10	53.9	14.5	<10	71.0	14.2	<10	85.5	64.1	>80	<10		<10	<10	<10		<10	<10
HaCa T	<10	<10	<10	>SO	>80	46.6	62.1	<10	<10	>80	>80	>SO	10.3		<10	<10	18.1		<10	<10
T24	<10	<10	<10	>80	53.0	22.5	66.7	<10	<10	>80	>80	>80	<10		<10	<10	<10		<10	<10
NC1–H226	60.3	<10	<10	55.9	19.9	<10	60.6	11.8	<10	68.7	45.0	21.2	<10		<10	<10	<10		<10	<10
MIA-PA-CA -2	<10	<10	<10	54.5	29.8	<10	54.2	25.1	<10	75.3	52.3	29.4	<10		<10	<10	<10		<10	<10
SCC-29 ​B	NE	<10	<10	69.8	43.8	17. 8	12	<10	<10	>80	>80	>80	<10		<10	<10	<10		<10	<10
HEK-293	<10	<10	<10	>80	53.0	21.4	>80	<10	<10	>80	>80	>80	<10		<10	<10	NE		<10	<10
SCC-40	<10	<10	<10	>80	59.2	24.8	>80	46.2	<10	>80	>80	>80	<10		<10	<10	<10		<10	<10
U373MG	<10	<10	<10	>80	52.1	19.4	63.7	<10	<10	>80	>80	>80	<10		<10	<10	<10		<10	<10

### 3.2 Molecular docking studies

The molecular interaction studies of the selected targets with ligand SERMS were carried out based on the genes mentioned in the materials and methodology section. The Molecular properties and toxicity of the each drug molecule was calculated through Discovery studio 2018 and Adriamycin was found to be mutagen while other SERMS were non-mutagenic **(**Table 3). The toxicity of each drug molecule was calculated based on the 2D structure of the molecules through TOPKAT. The program assess the toxicity based on Ames mutagenicity which help to characterize the molecule as mutagenic or non-mutagenic. The molecular properties of the drug molecule was characterized based on Lipinski's rules. Molecular weight and A Log P of all SERMS were predicted, of these adriamycin with 543.51 ​Da (>500) as its molecular weight and A Log P for Doxorubicin was −0.044 (<5) with violations. But for the hydrogen bond donor and acceptor, the values were equal to or less than 5 for all SERMS where for Adriamycin the value was 6 and 12 respectively.

**Table 3:** Molecular properties and toxicity of the each drug molecule; calculation can be done through Discovery studio 2018.

Sl No	Ligands	Molecular Weight (Daltons)	A Log P (Water partition co-efficient)	No: of H-bond donor	No: of H-bond acceptor	Toxicity
1.	Bazedoxifene	470.60	7.22	2	4	Non-mutagen
2.	Adriamycin	543.51	−0.044	6	12	Mutagen
3.	Dasatinib	488.00	3.43	3	**8**	Non-mutagen
4.	Gefitinib	446.90	4.20	1	7	Non-mutagen
5.	Olaparib	434.46	2.12	1	4	Non-mutagen
6.	Ormeloxifene	457.60	6.12	0	4	Non-mutagen
7.	Pepstatin A	685.89	2.30	**8**	9	Non-mutagen
8.	Raloxifene	473.58	6.46	2	5	Non-mutagen
9.	Tamoxifene	371.51	6.31	0	2	Non-mutagen
10.	Vemurafenib	489.92	4.95	2	4	Non-mutagen
11.	Vorinostat	264.32	2.00	3	3	Non-mutagen

Table 4 illustrates the docking score in Kcal/mol of each ligand with the targets, its hydrogen bond interaction with critical residues and the bond distance. The drug Ormeloxifene showed better binding interaction with target proteins such as 3ERT (ER-alpha), 5IWG (HDAC-2), 5UGA (EGFR kinase), 4MXO (EGFR cSRC), 5WS1 (PARP-1), and 2FB8 (BRAF) compared to that of tamoxifene. The drug Ormeloxifene had interaction with Asp351 residue of Estrogen receptor alpha (3ERT) with docking score of 119.24 ​kcal/mol than that of tamoxifene with score of 105.36 ​kcal/mol. The standard drug of HDAC2 (5IWG) is vorinostat have binding affinity with critical amino acid residues such as Gly142, and Gly154, the drug Ormeloxifene binds at the critical amino acid residues Gly154 with docking score of 130.76kcl/mol., Tamoxifene did not show any interaction with critical amino acid residue of HDAC2. Ormeloxifene showed two Hydrogen bond interaction with Met793 and one with Cys797 critical residues of Epidermal growth factor receptors (5UGA) with docking score of 132.25 ​kcal/mol higher than that of Standard drug Gefitinib. In Poly [ADP-ribose] polymerase 1, showed better binding with Ormeloxifene with interaction of Asp766 one of the critical residues with score of 159.29 ​kcal/mol than that of Tamoxifene with 128.69 ​kcal/mol. The drugs such as Ormeloxifene, Tamoxifene and standard drug showed exact binding at critical amino acid residue of cys532 of BRAF with docking score of 118.95, 99.161, 138.27 ​kcal/mol respectively. The drug ormeloxifene was very much comparable with standard drug at its binding amino acid and also in terms with docking score. The proteins such as Poly [ADP-ribose] polymerase 2, Cathepsin D, and Hsp70 had no critical amino acid interaction with Ormeloxifene. The dug Bazedoxifene had high affinity and docking score with all the target proteins. The drug Ormeloxifene showed better binding interaction with target proteins such as 3ERT (ER-alpha), 5IWG (HDAC-2), 5UGA (EGFR kinase), 4MXO (EGFR cSRC), 5WS1 (PARP-1), and 2FB8 (BRAF), 3KJD (PARP-2), 5ITA (BRAF kinase), 4OD9 (Cathepsin) and IUY8 (HSP-90) compared to that of tamoxifene with better docking scores (Kcal/mol) (highlighted red in Tables 4A and 4B. But compared to the positive control the docking score of Ormeloxifene is lower, yet comparable for the target proteins 3KJD (PARP-2), 2FB8 (BRAF), 5ITA (BRAF kinase) and 4OD9 (Cathepsin D). The docking scores of Ormeloxifene were comparable with Adriamycin also for all target proteins used for the studies. The docking score of Ormeloxifene and Tamoxifene are indicated in red colour to get a good comparison in Table 4. Fig. 3A, Fig. 3B, Fig. 3C illustrates the comparison of 2D interactions of the drug Ormeloxifene and Tamoxifene with targets 4MXO, 5WS1 (Fig. 3A) 3ERT, 5IWG (Fig. 3B), 2FB8 and 5UGA (Fig. 3C). The 3D images of the protein-ligand interactions of Ormeloxifene with the ligands is presented as 4MXO, 5WS1 (Fig. 4A), 3ERT, 5IWG (Fig. 4B), 5UGA, and 2FB8 (Fig. 4C) The interaction of drug molecules with the targets at its active site were represented either by 2D or 3D.The drug molecules that interacts with critical amino acids were taken, and others were excluded. Based on these observations, ormeloxifene was found to be an effective promising SERM which could be exploited for further studies, compared to other SERMS especially Tamoxifene and Hydroxytamoxifene currently used for treatment of breast cancer

**Table 4:** Docking score in Kcal/mol of ligand Vs target of each gene and its Hydrogen bond interaction with bond distance in A^0^.

Table 4A Docking score, Hydrogen bond interaction, Bond distance (A^O^) of the ligands with ER- α, HDAC-2, EGFR kinase, EGFR, PARP-1).					
Sl:No.	Target with PDB ID	Ligands	Docking score Kcal/mol.	Hydrogen bond interaction	Bond Distance A^0^
1	ER- ​α 3ERT	Bazedoxifene	145.70	Gly 420(CHB), Gly521,Leu346,Leu525	2.20 2.70,3.08,2.18
		Adriamycin	125.47	Trp 383,Ser 518(2),Asn 519, Glu 380,	2.31,2.80,2.13,2.45,2.73
		Raloxefene	124.87	His 524	2.29
		Ormeloxifene	119.24	Asp 351(SHB), Gly521(2),Gly420	2.03, 1.92,2.68,2.29
		Tamoxifene (Std. drug)	105.36	Asp 351shb, Asp351whb	2.00,2.85
2	HDAC2 5IWG	Bazedoxifene	146.03	Gly 154	2.67
		Adriamycin	89.19	Cys105,Gly154,Asn 100(2)	
		Raloxefene	133.61	Tyr308,Gly143,Asp181,Gly154	2.08,2.33,2.71,2.54
		Ormeloxifene	130.76	Gly 154, Tyr 308	1.67,2.39
		Tamoxifene	77.45	Gly 154	2.31
		Vorinostat (Std. drug)	127.03	Gly 305, Gly 154, Gly 142	2.88,1.71,1.65
3	EGFR Kianse 5UGA	Bazedoxifene	131.00	Met 793, Phe 795	2.50,1.86
		Adriamycin	137.41	Met 793, Gln 791	2.86,2.12
		Raloxefene	122.17	Gln 791, Met 793	2.20,1.95
		Ormeloxifene	132.25	Cys 797, Met 793	2.95,2.29
		Tamoxifene	98.594	Met 790	2.50
		Gefitinib (Std. Drug)	106.21	Met 793	2.67
4	EGFR cSRC 4MXO	Bazedoxifene	129.16	Asn391	2.72
		Adriamycin	138.30	Met 341, Asn 391, Asp 404	2.06,1.87,2.81
		Raloxefene	110.4	Ser345, Asp404, Gly279	2.03,2.31,2.48
		Ormeloxifene	118.8	Met 341, Thr 338	2.23,1.69
		Tamoxifene	93.43	Met341	2.44
		Dasatinib (Std. Drug)	122.79	Tyr 340 Met 341, Thr 338	2.96,2.12,2.15
5	PARP-1 5WS1	Bazedoxifene	178.95	Asn 767, Gly 888, Asp 766	2.68, 2.75,2.83
		Adriamycin	172.52	Glu 763, Thr 887, Glu 988	1.92,2.86, 3.03
		Raloxefene	164.08	Gly 888, Ala 898	2.88,2.91
		Ormeloxifene	159.29	Asn 767(2) Asp 766.	2.58,2.758,2.87
		Tamoxifene	128.69	Tyr 907	2.42
		Olaparib (Std. Drug)	168.07	Gly 863, Arg 878(2)	2.57,1.91,2.69
Table 4B Docking score, Hydrogen bond interaction, Bond distance (A^O^) of the ligands with PARP-2, BRAF, BRAF kinase, cathepsin, HSP-90).					
6	PARP-2 3KJD	Bazedoxifene	178.32	Met 456, Gly 454, Arg 444	2.04,2.71,2.53
		Adriamycin	167.66	Ser 470, Tyr 473(2)	2.11,2.95,2.28
		Raloxefene	169.14	Ile 445, Gly 454, Arg 444.	2.30,2.51,2.30
		Ormeloxifene	163.41	Met 456	1.96
		Tamoxifene	131.10	Gln 332, Gln 335	2.85,2.84
		Olaparib (Std. Drug)	165.86	Gly 429	2.68
7	BRAF 2FB8	Bazedoxifene	142.15	Glu 533	2.30
		Adriamycin	120.39	Cys 532, Asp594, Asn581,	1.75,2.20,2.43
		Raloxefene	119.83	Gln 531,Thr529	2.69,2.88
		Ormeloxifene	112.54	Cys 532	1.84
		Tamoxifene	99.161	Cys 532,Gly534	2.85,2.97
		Vemurafenib (Std. Drug)	138.27	Cys 532	1.59
8	BRAF Kinase Domain 5ITA	Bazedoxifene	133.94	Ser 465, Gly 534	2.32,1.89
		Adriamycin	152.39	Cys 532(3)	2.62,2.49,1.41
		Raloxefene	121.01	Lys 483, Gly 466	2.98,2.05
		Ormeloxifene	122.64	Lys 483	2.48
		Tamoxifene	105.25	Asp 594	1.81
		Vemurafenib (Std. Drug)	151.51	Lys 483, Gly 596, Phe 595, Gln 530	2.36,2.07,2.47,1.56
9	Cathepsin 4OD9	Bazedoxifene	100.83	Gly79	2.38
		Adriamycin	118.98	Ser80, Ser235, Leu236, Gly233	2.33,2.26,2.79,2.76
		Raloxefene	76.97	Ala128	2.08
		Ormeloxifene	125.88	Val41, Asp90.	
		Tamoxifene	80.22	Asp 33 whb	2.36
		Pepstatin A (Std. Drug)	161.49	Ser 80, Gly 233,	2.77,2.21
10	HSP-90 1UY8	Bazedoxifene	149.82	Ser 52	2.62
		Adriamycin	138.51	Asn 51, Gly 137, Phe138, Asp 54, Tyr 139, Glu 47, Gly 132	1.94,2.26,2.39,1.80,2.30, 2.58,1.84
		Raloxefene	135.93	Asp 93	2.62
		Ormeloxifene	132.21	Gly 135	2.02
		Tamoxifene	107.83	Leu 103	2.11
		Ganetespib	113.90	Asp 93	2.0

**Fig. 3A:**
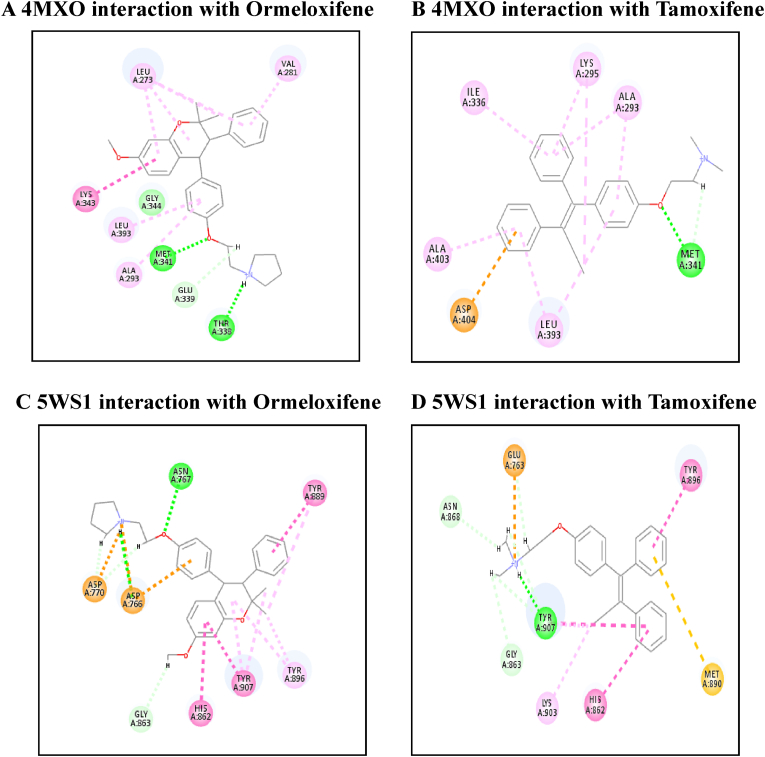
2D representation of molecular interaction of ormeloxifene and tamoxifene with targets 4MXO and 5WS1. D 5WS1 interaction with Tamoxifene. C 5WS1 interaction with Ormeloxifene. B 4MXO interaction with Tamoxifene. A 4MXO interaction with Ormeloxifene. 2D Interaction of ormeloxifene and tamoxifene with its critical amino acid residues in the receptors with (A,B) 4MXO (C,D) 5WS1.

**Fig. 3B:**
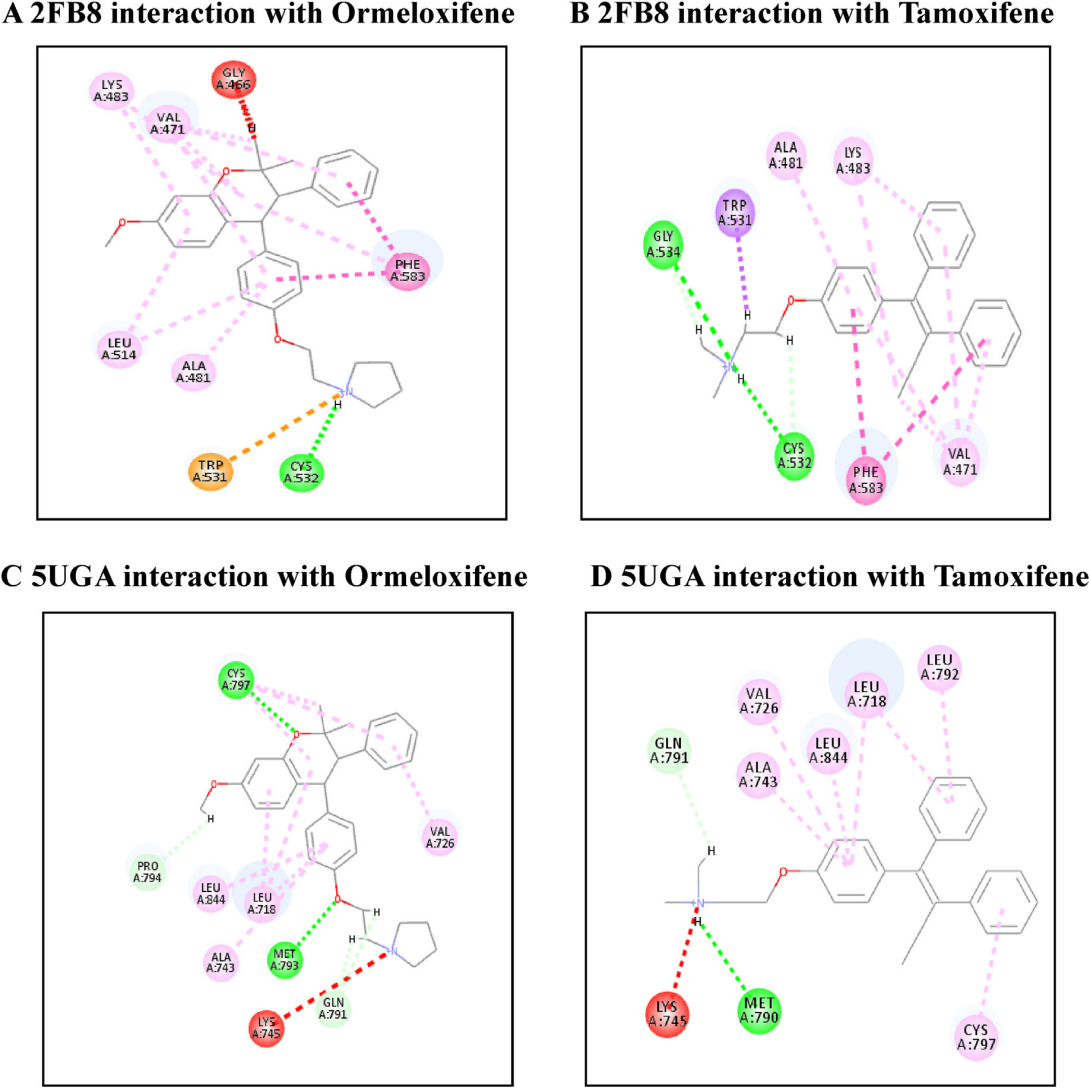
2D representation of molecular interaction of ormeloxifene and tamoxifene with targets 2FB8 and 5UGA. D 5UGA interaction with Tamoxifene. C 5UGA interaction with Ormeloxifene. B 2FB8 interaction with Tamoxifene. A 2FB8 interaction with Ormeloxifene.2D Interaction of ormeloxifene and tamoxifene with active site amino acid residues of the receptors (A,B) 2FB8 (C,D) 5UGA.

**Fig. 3C:**
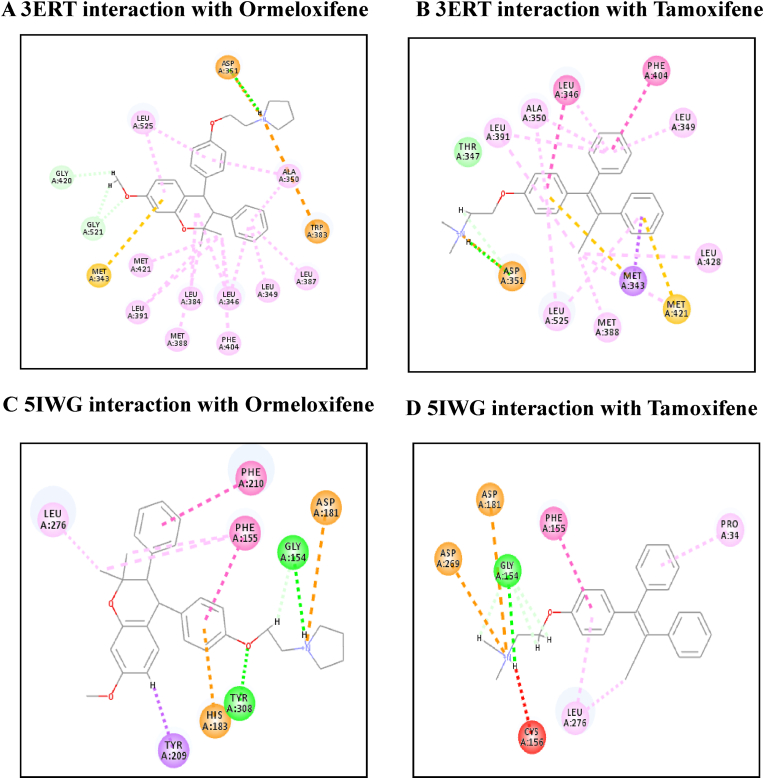
2D representation of molecular interaction of ormeloxifene and tamoxifene with targets 3ERT and 5IWG. D 5IWG interaction with Tamoxifene. C 5IWG interaction with Ormeloxifene. B 3ERT interaction with Tamoxifene. A 3ERT interaction with Ormeloxifene. 2D Interaction of ormeloxifene and tamoxifene with active site amino acid residues of the receptors (A,B) 3ERT (C,D) 5IWG.

**Fig. 4A:**
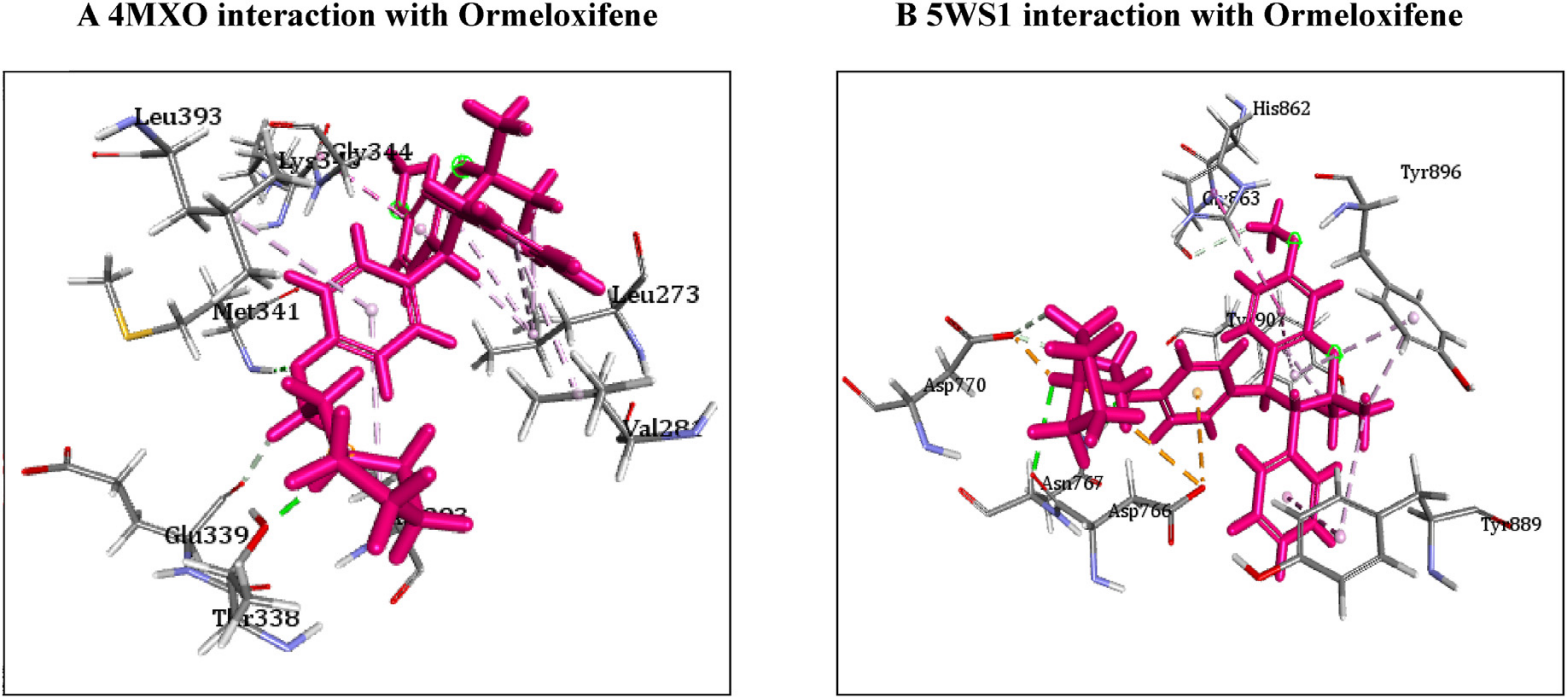
3D representation of molecular interaction of ormeloxifene with targets Interaction of ormeloxifene with receptors 4MXO and 5WS1. B 5WS1 interaction with Ormeloxifene. A 4MXO interaction with Ormeloxifene. 3D Interaction of ormeloxifene with active site amino acid residues of the receptors (A) 4MX0 (B) 5WS1. Ormeloxifene interacted with its active amino acids in the pockets EGFR cSRC and PARP-1respectively.

**Fig. 4B:**
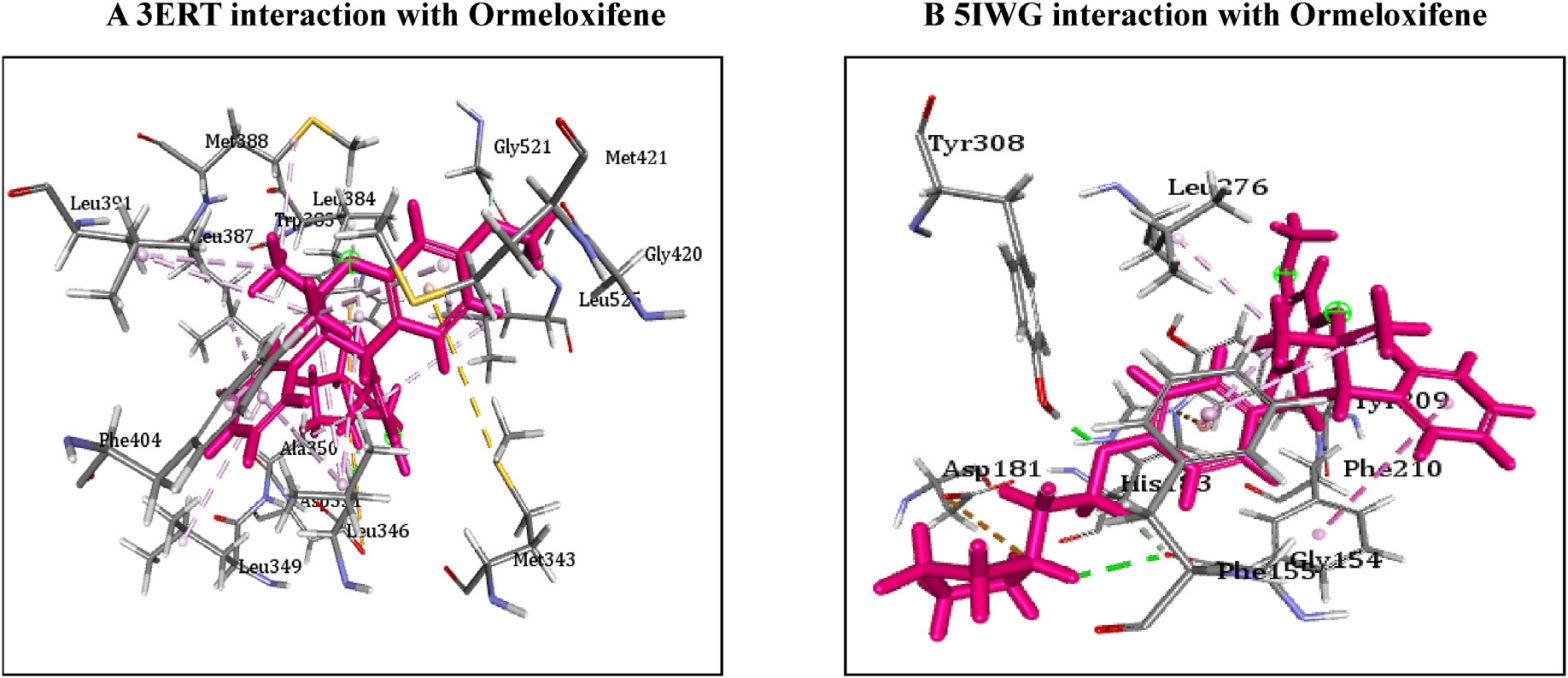
3D representation of molecular interaction of ormeloxifene with targets Interaction of ormeloxifene with receptors 3ERT and 5IWG. 3D Interaction of ormeloxifene with active site amino acid residues of the receptors (A) 3ERT (B) 5IWG. Ormeloxifene interacted with its active amino acids in the pockets ERα and HDAC2 respectively.

**Fig. 4C:**
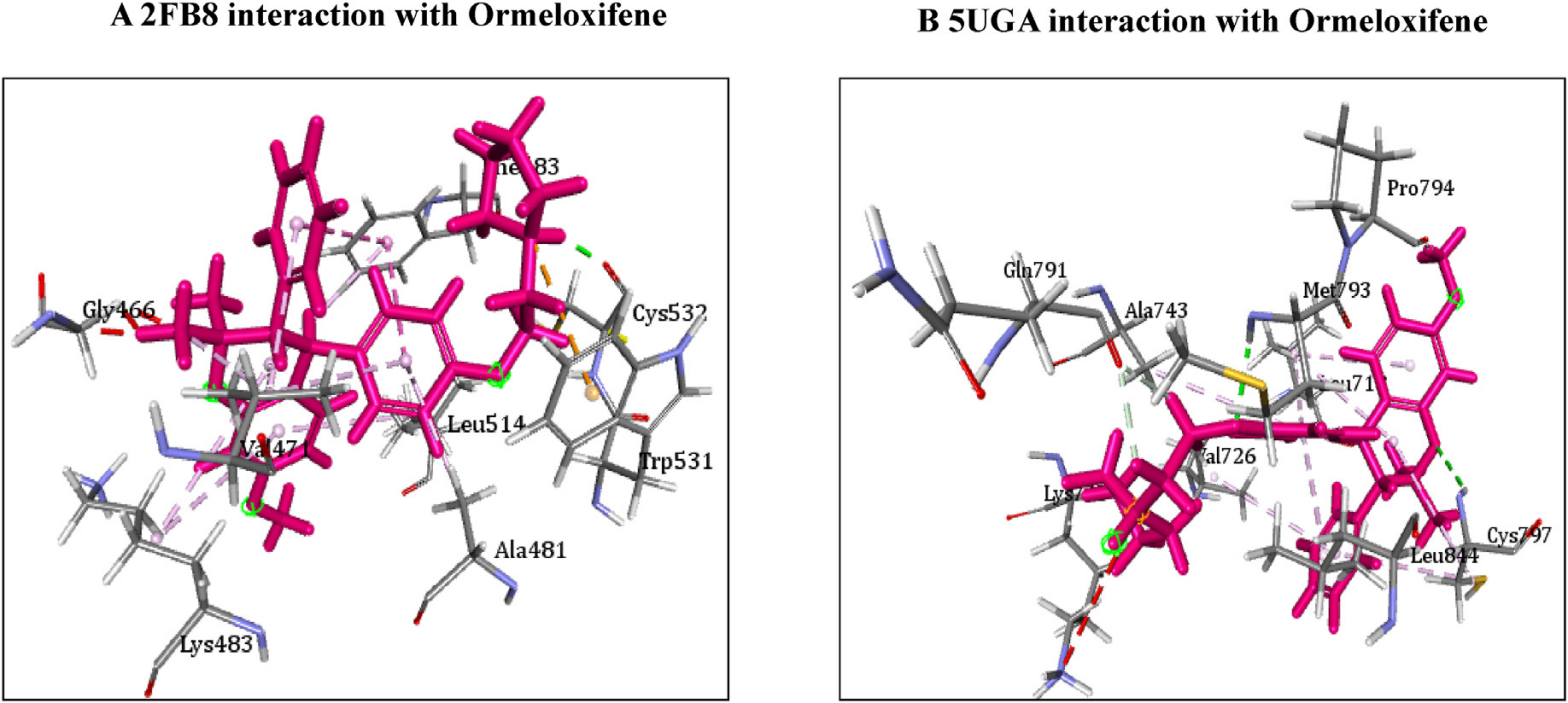
3D representation of molecular interaction of ormeloxifene with targets Interaction of ormeloxifene with receptors (A) 2FB8 and 5UGA. 3D Interaction of ormeloxifene with active site amino acid residues of the receptors (A) 2FB8 (B) 5UGA. Ormeloxifene interacted with its active amino acids in the pockets BRAF and EGFR kinase respectively.

## 4 Discussion

SERMS are drugs that activate the estrogen receptors and have different effects on different tissues. There are two kinds of estrogen receptors, and after binding to receptors, the drug-receptor complex could possess various conformations. Some SERMS bind to estrogen binding receptor and inhibit the harmful actions of estrogen in tissues like breast thus decreasing the risk of breast cancer and other hormone related disorders. In another case, they act estrogenic in tissues like bones and ovary, thus protecting these organs. Scientists are constantly in search for SERMS that could stabilize bone mass, improve lipid profile and reduce hot flashes, at the same time, which are anti-estrogenic for treating breast cancer and lesser stimulation of the endometrium which leads to endometrial cancer.

Tamoxifen is a first line SERM that is being currently used as gold standard to treat breast cancer. It has some beneficial effects on the bones, but its long term usage has some predisposition to endometrial cancer and some other side effects owing to the depletion of estrogen. Tamoxifen has been estimated to have saved the lives of about 400,000 women who have suffered with breast cancer (Y Maximov et al., 2013) The second generation SERM, raloxifene failed as a treatment for breast cancer but was reported to be effective for treatment of osteoporosis and prevention of breast cancer at the same time. Also raloxifene was reported to reduce invasive breast cancer risks without an increase in the risk of endometrial cancer observed with tamoxifen (Cummings et al., 1999). Another study suggested that raloxifene might even be effective in preventing endometrial cancer (DeMichele et al., 2008). Tamoxifen molecule is required to be hydroxylated and demethylated to form the active metabolites 4-hydroxytamoxifen and endoxifen, mutations in the CYP2D6 gene impair tamoxifen's efficacy to form the active metabolites (Goetz et al., 2008). Toremifene is a chlorinated tamoxifen analogue which has been approved in the US and several other countries for the treatment of metastatic breast cancer. Toremifene is as effective as tamoxifen in the treatment of ER-positive breast cancer However, there are some reports to show toremifene induces DNA damages and hepatocarcinogenesis in rats (Dragan et al., 1995; Sargent et al., 1996).

Centchroman or Ormeloxifene is reported to possesses excellent therapeutic index and has been well tolerated, without any haematological, biochemical or histopathological evidence of toxicity when administered at many times the contraceptive dose (Kamboj et al., 2018; Singh, 2001). Ormeloxifene is reported to treat several hormonal indications. A randomized trial to evaluate effectiveness of Centchroman in control of mastalgia in comparison with Danazol was carried out by All India Institute of Medical Science, Delhi. Eighty one patients were evaluated with breast pain. Treatment with Ormeloxifene has been evaluated in 81 patients. The overall response rate was 89.7% at 12 weeks of therapy. At 24 weeks follow up, the response rate in Centchroman and Danazol was 71.05%, 42.42% respectively (Dhar and Srivastava, 2007; Yasemin and Mehmet, 2019). Ormeloxifene is reported which could be used in the management of abnormal uterine bleeding where uterus size is not very big and could avoid many hysterectomies. Long-term study and meta-analysis have already proved its safety and efficacy that the drug is equally effective in premenopausal women of all age groups (Pati et al., 2017; Mani et al., 2019; Vardaini et al., 2020). Fibro adenomas are one of the most common benign tumours of the breast in women under 30 years of age and account for 68% of all breast masses and 44%–94% of biopsied breast lesions in which ormeloxifene has been reported to have good responses (Tejwani et al., 2015). Ormeloxifene is reported to be a good candidate for treatment of osteoporosis as evidences from osteoclast differentiation studies in rats (Murthy et al., 2006).

Ormeloxifene is reported to be a potent non-steroidal agent that has been widely shown to act upon several important molecular targets in cancer cell lines. The survey of literature also suggests that it has an excellent therapeutic index and is considered safe for chronic administration. Moreover, the molecule is reported to exert anti-tumor activity independent of estrogen regulation, exerting its effects via HDAC inhibition, down regulation of tumor promoter genes, human telomerase reverse transcriptase and expression of tumor suppressor genes which explains genetic and epigenetic modes of action which is very important in anti-cancer therapy (Nigam et al., 2010; Nigam et al., 2008; Srivastava et al., 2011; Molinie and Georgel, 2009; Kachchap et al. 2010; Agrawal et al., 2016a; Khan et al., 2016; Rama Raju et al., 2015; Khan et al., 2015; Singh et al. 2016; Mishra et al., 2010; Mukhopadhyay et al., 1999; Mukhopadhyay et al., 2001; K. Giri et al. 1999; Dewangan et al., 2018; Bhattacharjee et al., 2018; Zhang et al., 2012; Park et al., 2008; Pati et al., 2017).

Estrogen receptors are present at the cell membrane to be part of the rapid phosphorylation signal transduction mechanism and part of the mitochondrial mechanisms for cell survival (Mendez et al., 2006). The ligand structure is also important to cause distinct ER conformations that will in turn affect the subsequent interactions with coactivators or corepressors. The SERM-ER complex so modified by interaction with coactivators could enhance gene transcription and corepressors that could reduce gene expression (Smith and O'malley 2004). The molecular docking studies done with the important clinically used SERMS provide a valuable insight into the molecular interactions between the ligand and protein. Each time these computational methods verify already established experimental results; their validity in the drug design market has the opportunity to go up. This is a comprehensive research work done to identify and compare the effective concentrations of five different SERMS in comparison with Adriamycin, a standard aromatase inhibitor class of compound on twenty six different cancer cell lines. Ormeloxifene showed good differential cytotoxic effects than other SERMS especially Tamoxifene and its active metabolite 4-hydroxy Tamoxifene and raloxifene in almost all cell lines which proves its efficacy to be exploited further. The GI_50_ value was observed to be ​< ​10 ​μg/ml using Ormeloxifene whose effects were very comparable with that of endoxifene and adriamycin. But the concentrations for TGI and LC_50_ were higher for endoxifene and Adriamycin compared to Ormeloxifene in some of the cancer cells lines. In light of this, a predictive bioinformatics based study was done using computational approaches in the key target genes regulated by SERMS. The targets were selected on the basis of its regulation in cancer progression and treatment responses. Good binding score was obtained using Ormeloxifene with estrogen receptor regulated ER-α, EGFR kinase and EGFR-C-src and also with HDAC-2, PARP-1 and BRAF ligands compared with Tamoxifene which shows the efficacy of the compound.

HDACs are reported to induce a range of cellular and molecular effects through hyper acetylation of histone and non-histone substrates. HDACs is reported to either repress tumor suppressor gene expression or regulate the oncogenic cell-signalling pathway via modification of key molecules and have shown to regulate apoptosis by regulating expression of pro and anti-apoptotic proteins in cancer cells (Li and Seto, 2016). BRAF is reported to stimulate ERK signalling, induces proliferation and is capable of promoting transformation and taken into consideration the aberrant mutations in BRAF, efforts are underway to develop targeted inhibitors of BRAF and its downstream effectors (Pratilas et al., 2010). c-Src and EGFR have been shown to enhance pro-mitogenic signals upon epidermal growth factor (EGF) stimuli (Luttrell et al., 1988) c-Src and activated EGFR cooperate to induce cell transformation and cancer development (Maa et al., 1995) by binding to EGFR and thus phosphorylating tyrosine residues on its C-terminal domain, resulting in a variety of downstream effects. c-Src activation induced by EGFR ligands mediates the binding of phosphatidylinositol 3-kinase (PI3K) to EGFR, leading to AKT phosphorylation and, in turn, induction of survival and migration signalling pathways (Franke et al., 2003; Shien et al., 2004; Jiang et al. 2003). PARP1 maintains cellular functions, including DNA repair/maintenance of genomic integrity, DNA methylation, chromatin regulation, and histone modification and helps in the recruitment of HDAC1 and HDAC2 as chromatin modifications in cancer (De Vos et al., 2012; Kraus, 2008).

The anti-cancer effects and the cytotoxic responses in this study in various cancer cells by the SERM, Ormeloxifene might be due to the regulation of these key target genes which could regulate cancer progression and apoptotic responses. More studies are warranted to identify more target genes in the process of cancer progression and treatment responses which are required to confirm with gene expression studies in these cell lines.

## 5 Conclusions

SERM class of compounds have been used for treating breast cancer, osteoporosis and postmenopausal symptoms, as these could act as both an estrogen agonist and an antagonist, depending on the target tissue. After tamoxifen, raloxifene, 5-hydroxy tamoxifene and endoxifene and other SERMS have been developed and used for treatment. The clinically decisive difference among these drugs mostly depends upon their endometrial safety. Research on adverse effects of SERMS agents is being studies throughout to determine the long-term safety of this class of compounds for treatment. Ormeloxifene is a SERM class of compound currently used contraception, treatment of mastalgia, abnormal uterine bleeding and fibro adenoma. The studies prove that the compound is well tolerated for long term safe usage without endometrial and other hormonal complications. Also it was found to have good cytotoxic effects on almost twenty six different cancer cell lines irrespective of the hormonal regulation which proves that it could act in both hormonal and hormonal independent cancers. The computational studies also prove that the compound could regulate the key pathways in cancer progression and development and apoptosis. The studies put forth the efficacy of Ormeloxifene to be repositioned as an anti-cancer drug and the need for more studies on the mechanism of action of Ormeloxifene.

## Availability of data and material

The authors declare that all data presented are publicly available upon request.

## Code availability

Not Applicable.

## Authors contribution

All authors have contributed equally in the research as well the Framing of the Manuscript.

## Ethics approval

Not Applicable.

## Consent to participate

Not Applicable.

## Consent for publication

All authors approve of this submission for publication.

## Declaration of competing interest

The authors declare that they have no known competing financial interests or personal relationships that could have appeared to influence the work reported in this paper.
